# Physiological Response of Crimson Seedless Table Grape Vines to Controlled Irrigation Conditions in Different Micro-Climatic Environments

**DOI:** 10.3390/plants14233579

**Published:** 2025-11-23

**Authors:** Janéne Strydom, Cornelis G. Volschenk, Marieta van der Rijst, Laura de Palma, Vittorino Novello, Rosario di Lorenzo, Antonino Pisciotta, Pieter J. Raath, Jacobus J. Hunter

**Affiliations:** 1 Crop Development Division, ARC Infruitec-Nietvoorbij, Private Bag X5026, Stellenbosch 7599, South Africa; volschenkn@arc.agric.za; 2 Biometry, ARC Infruitec-Nietvoorbij, Private Bag X5026, Stellenbosch 7599, South Africa; vanderrijstm@arc.agric.za; 3 Department of the Science of Agriculture, Food and Environment, University of Foggia, Via Napoli, 25, 71122 Foggia, Italy; laura.depalma@unifg.it; 4 Department of Agricultural, Forest and Food Sciences (DiSAFA), University of Turin, Largo P. Braccini, 2, 10095 Turin, Italy; vittorino.novello@unito.it; 5 Department of Agricultural and Forest Sciences, University of Palermo, Viale delle Scienze 11, 90128 Palermo, Italy; rosario.dilorenzo@unipa.it (R.d.L.); antonino.pisciotta@unipa.it (A.P.); 6 Department of Horticultural Science, Stellenbosch University, Private Bag X1, Stellenbosch 7602, South Africa; pjraath@sun.ac.za; 7 Independent Scientist, Private Bag X5026, Stellenbosch 7599, South Africa; hunterkobus@outlook.com

**Keywords:** temperature, relative humidity, soil water content, stem water potential, light intensity, photosynthesis, transpiration, water use efficiency, plastic cover

## Abstract

Producing table grapes in altered environments requires an understanding of grapevine physiological responses. This study aimed to determine the physiological reactions of Crimson Seedless/Ramsey vines in response to different water application levels in different micro-climates. Irrigation treatments entailing 100% (W100 = control), 80% (W080), 70% (W070), and 55% (W055) of control volumes were applied to open field (OF) vines and vines underneath overhead plastic covering (OPC). Soil water content (SWC), net photosynthesis (Pn), stem water potential (Ψ_S_), transpiration (Tr), micro-climatic variables, and leaf area were measured. The W080 and W070 treatments for OF and OPC, respectively, did not impair physiological processes compared to W100. Conversely, W055 significantly decreased Ψ_S,_ Pn, and Tr in both trials compared to W100. Underneath OPC, SWC, leaf area, Pn, and Tr values were higher than under OF conditions. Compared to OF, OPC decreased light intensity and increased Ψ_S_, albeit resulting in lower photosynthetic water use efficiency. Vines receiving W070 and W055 experienced less stress underneath OPC than under OF conditions, indicating that OPC improves response to water deficit conditions. Additionally, W055 under OPC lowered the average air temperature compared to OF. Water supply reductions of 20% under OF conditions and 30% underneath OPC sufficiently maintained physiological processes.

## 1. Introduction

Changes in photosynthetic activity, stomatal movement, internal CO_2_ concentration, transpiration, and stem water potential are related to plant water relations [1,2]. Stomatal movement regulates water loss [1,2] and is dictated by environmental conditions, such as soil water availability [3]. Responses to water deficits become more pronounced when water deficit conditions are combined with high vapour pressure deficits [4], high light intensities [5], and high temperature conditions [6].

In a specific micro-climatic environment, the atmospheric temperatures surrounding the leaves affect leaf water potential and gas exchange [7]. Above 35 °C, photosynthesis may decline [6]. The negative effects of high temperatures on photosynthesis can be mitigated by water availability since higher stomatal conductance and transpiration rates associated with high temperature enhance leaf cooling when root water absorption is not limited [8,9]. In this regard, increased photosynthetic rates have been reported for sun-exposed leaves of irrigated Shiraz vines in an enclosed field chamber at a temperature of 40 °C for three days [10]. In cooler regions, with temperatures between 15 °C and 25 °C, stomatal closure and photosynthesis can be restricted [11].

The severity of water deficit conditions determines grapevine physiological responses and can be classified as mild, moderate, and severe [12,13]. However, responses may vary under the same conditions for different cultivars [14]. Stomatal closure is an initial response to water deficit conditions, which influences photosynthesis [1,2,12]. In addition to stomatal responses, decreases in photosynthetic rates under moderate water stress conditions can be ascribed to non-stomatal responses, such as carbohydrate accumulation [15]. Severe water deficit conditions cause a sharp decline in CO_2_ assimilation, electron transport rate, and carboxylation, whereas non-photochemical quenching increases [12] and Rubisco activity becomes inhibited [14]. However, the down regulation of Rubisco activity is not easily proven under field conditions [16].

Generally, under mild water deficit conditions, elevated CO_2_ levels increase photosynthetic activity and enable the use of CO_2_-induced carbohydrates for structural growth [15,17]. Elevated CO_2_ may lead to photosynthetic acclimation in the long-term [15,18].

Manipulation of the micro-climatic environment is a means to buffer table grape vines against environmental conditions. Alternative cultivation practices, such as cultivation under protection, in addition to/as opposed to standard cultivation practices, may be used to achieve this goal. The purpose of cultivation determines the time of plastic cover installation. When vineyards are protected with overhead plastic covers directly after accumulation of the required number of chill units, budbreak, and thus ripening, are enhanced [19]. Late covering, at the start of ripening, is a technique adopted to delay the harvest [19]. Vines can be protected against adverse environmental conditions throughout the entire growing season by covering the vineyard just after budbreak and then removing the covers when the leaves fall [19].

The use of overhead protective covers is a general practice in many table grape cultivation areas, especially where environmental conditions, such as rain, pose threats for losses during the ripening period [20,21]. Covering vineyards with thin protective plastic films during ripening protects harvests against rain and diseases [19,22].

Apart from a protective function, overhead plastic covering (OPC) can temper micro-climatic conditions by decreasing temperature and increasing humidity, ensuring conditions for plant performance to be more favourable for phenolic and total soluble solids accumulation [23]. Compared to open field (OF) conditions, OPC contributes to lower evapotranspiration [24] due to lower radiation reaching the soil surface [25], thus preserving the soil water content (SWC). Da Silva et al. (2018) [26] reported a water saving when the top sides of Niagara Rosada grapevines were covered with transparent plastic covers. The light spectrum underneath OPC is determined by the radiometric properties of the plastic product [27]. The radiometric properties of the products used for overhead covering are vital because of the light conditions inside the canopy and around the bunches that facilitate plant productivity by activating genes and enzymes [28,29] through a thermal or a phytochrome effect [30]. Ideally, the light environment conditions must allow for approximately 60% of phytochrome to be in the far-red form [31] (Smith, 1982) because of its role in the activation of, e.g., nitrate reductase [30,32]. Overhead plastic covers with high transmissivity for photosynthetic active radiation (PAR) ensure optimal photosynthesis [33,34], if temperatures do not increase beyond 35 °C and water is available [33,35]. Material with a solar transmissivity of at least 80% is required to ensure adequate illumination of the canopy to allow its regular functioning; furthermore, the solar transmissivity of the plastic sheet is partly responsible for the temperature of the confined environment [20,21]. Transmittance for ultraviolet (UV) light favours production of secondary compounds, such as phenols, volatiles, and carotenoids [28,36], whereas transmittance of excessive UV-B fluence rates destroys proteins [37]. Low transmissions of long-wave infrared ensure R/FR ratios favouring the active form of phytochrome for the activity of various enzymes necessary to produce secondary compounds [30,31,32]

Local studies have defined ranges for the degree of water deficit in terms of Ψ_S_ for various table grape cultivars grown under open field conditions [38]. The limited available scientific information on physiological processes, such as Pn, Tr, and Ψ_S_, of table grape vines in response to drought and other micro-climatic conditions, such as altered temperatures and RH around the leaves and bunches under South African conditions, hinders technological advancement and thus efficient management of growth. Limited water supply and changes in the climate create challenging conditions, which require an understanding of the physiological status of table grape vines. Understanding plant responses to altered environmental conditions is critical to develop tools for regulatory management of growth balances under such challenging conditions.

The study aimed to (1) determine grapevine reaction to three levels of reduced water supply compared to the on-farm treatment, which was the amount of water needed in terms of crop evapotranspiration, and (2) determine grapevine reaction during the growth season to the four water supply treatments in OF conditions, and in conditions where the micro-climatic environment had been changed by means of OPC. The objectives of the study were to (1) obtain a better understanding of the vine reactions in terms of the SWP, Pn, and Tr under conditions of altered water supply and different micro-climatic environments (OF and OPC), and (2) provide producers with evaluation and measurement tools related to SWP and responses related to photosynthesis.

## 2. Results

### 2.1. Soil Characteristics

The soil physical and chemical characteristics in the OPC trial were relatively homogenous compared to those of the OF trial (Table 1). At a depth of 30 cm, in the OF trial, the clay content in the W070 plots was significantly more than the clay content in the W080 plots, whereas the silt content at 60 cm depth was significantly higher in the W070 plots compared to the plots for the other treatments. Compared to the W080 treatment plots, the W070 plots had significantly less sand throughout the soil profile in the OF trial. The water-holding capacity (WHC) of the soil in the W070 treatment plots was significantly higher than that of the W100 and W080 plots at 30 cm and 60 cm soil depths in the OF trial. However, the differences were quite small.

The soil pH_KCl_ throughout both trials ranged from 5.5 to 6.5. Calcium base saturation rates at 30 cm depth W070 plots were significantly lower than in the W080 plots, whereas Mg base saturation rates in the W070 plots were significantly higher than in the W080 plots in the OF trial. In the OF trial, W070 experimental plots had significantly higher exchangeable K at 30 cm and 60 cm depths compared to the W080 plots.

### 2.2. Soil Water Content

**Figure 1:**
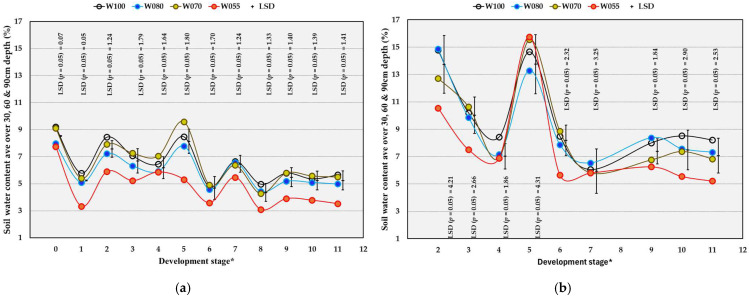
The effect of water treatments (W) on average soil water content over 30, 60 and 90 cm depth in the soil profile of a Crimson Seedless vineyard grown under open field (OF) conditions from 2019/2020 to 2022/2023 (**a**) and overhead plastic covering (OPC) from 2021/2022 to 2022/2023 (**b**). * Development stages: 0 = full bloom, 1 = berry set, 2 = 6 to 11 DAFB (Days after full bloom), 3 = 20 to 25 DAFB), 4 = 34 to 39 DAFB, 5 = 48 to 53 DAFB, 6 = 62 DAFB, 7 = 67 to 77 DAFB, 8 = 78 to 81 DAFB, 9 = 83 to 88 DAFB, 10 = 90 to 95 DAFB, 11 = 97 to 102 DAFB. W100 (100% = commercial practice), W080 (80% of commercial practice), W070 (70% of commercial practice), W055 (55% of commercial practice). LSD = Least significant difference.

In the OF trial, the W055 treatment significantly decreased the average soil water content (SWC) of the whole profile (30, 60, and 90 cm) compared to W100 at most of the development stages, except for DS 4, 6, and 7 (Figure 1a). From DS 2 towards the end of the harvest, the W070 treatment resulted in similar SWC to that of the W080 treatment, despite higher volumes of water applied in the latter (Table 2). In the OPC trial, the average SWC was decreased significantly by W055 compared to W100 at most of the development stages, except for DS 4, 5, 7, and 9 (Figure 1b). 

There were significant seasonal effects for the average SWC in both trials (Table S1). Soil water content in the OF trial was significantly higher in the 2022/2023 season than in the preceding seasons throughout the measurement period, at most of the development stages, except for DS 3, 4, and 7 (Table 3). In the 2022/2023 season, the average SWC in the OPC trial from DS 4 to 11 was significantly higher compared to the SWC in 2021/2022 (Table 3). Soil water content in the OF trial was lower than SWC in the OPC trial from DS 9 to 11 in the 2021/2022 and 2022/2023 seasons (Table 3). From DS 3 to 11 in 2022/2023, SWC in the OF trial was lower than in the OPC trial.

### 2.3. Climatic Conditions

**Figure 2:**
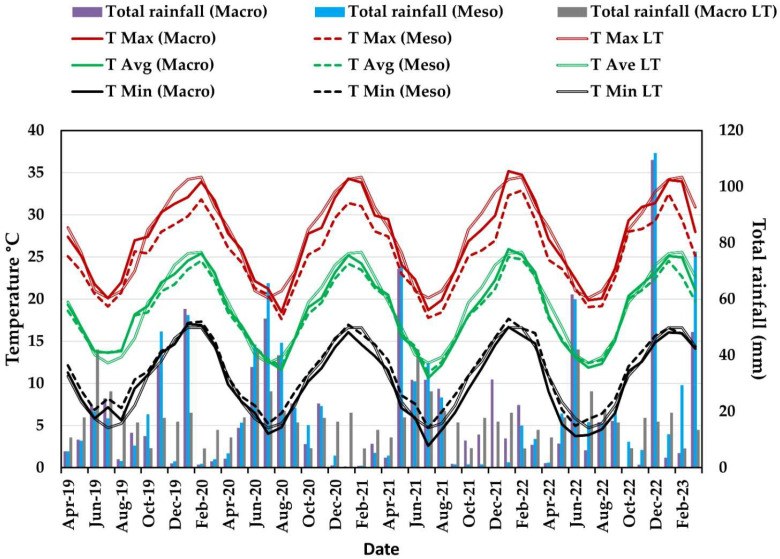
Temperature and rainfall in the macro- and meso-climatic environments near Robertson, South Africa, from April 2019 to February 2023, and LT (Long-term) temperature and rainfall in the macro-climatic environment (2013–2023).

Rainfall distribution during the ripening and harvest period was not the same in every season, and there were sporadic or erratic incidences of rainfall during the summer (Figure 2). January 2020 was characterised by the total rainfall exceeding 40 mm in the macro-climatic environment (MAC) and meso-climatic environment (MES). From December 2020 until February 2021, rainfall was less than 5 mm per month. In December 2021, almost 15 mm of rain was recorded in the MAC and none in the MES. In December 2022, the MES rainfall exceeded 100 mm. The rainfall in the MAC and MES deviated the most from the MAC long-term averages in January 2020, December 2021, February 2022, December 2022, and February 2023 (Figure 2). In January and February 2022, the MAC rainfall exceeded that of the MES. In January and February 2023, the total MES rainfall exceeded the MAC rainfall. The higher rainfall during the 2021/2022 and 2022/2023 seasons caused a higher SWC (Table 3). The SWC increases of more than 50% at DS 5 in both trials in the 2022/2023 season, compared to 2021/2022 (Table 3), were due to more than 100 mm of rainfall in the MES proximity in December 2022 (corresponding to DS 5) (Figure 2).

Temperature (T) variables fluctuated between the macro- (MAC) and meso-climatic environments (MES). In all the seasons, the T fluctuated just above or below the long-term average. The average temperatures (T_ave_) in the MAC exceeded the T_ave_ in the MES in all seasons (Table 4, Table 5 and Table 6). The MES T_ave_ was similar to the T_ave_ in the micro-climatic environments (MIC).

For the duration of measurement in all seasons, maximum temperatures (T_max_) followed a consistent trend, where MES T_max_ > MIC T_max_ and minimum temperatures (T_min_) in the MIC > T_min_ MES (Table 4, Table 5 and Table 6). 

There were some significant differences in the MIC for T variables between treatments during the trials, but no consistent pattern. In the 2020/2021 season, at DS 8 and 9, W055 significantly increased T_max_, T_min_, and T_ave_ compared to the control (Table 4). In the following seasons, there were no differences between W055 and W100 in the OF trial (Table 5 and Table 6). Data collected underneath OPC in the 2021/2022 and 2022/2023 seasons are presented only as average values because temperature variables between the treatments did not differ significantly (Table 5 and Table 6).

In 2021/2022 and 2022/2023, the W055 treatment underneath OPC continuously decreased the T_ave_, T_max_, and T_min_ compared to the same treatment in the OF trial, except for DS 9 in the 2021/2022 season, when T_max_ underneath the OPC tended to be higher. Minimum temperatures for all treatments in the OPC trial compared to the OF trial were decreased at all development stages except for DS 11 in the 2021/2022 season. With some exceptions, the T variables for the treatments in the 2021/2022 season tended to be lower underneath the plastic covers than in the OF. In the 2022/2023 season, the T variables tended to be higher underneath the OPC compared to the OF.

The relative humidity (RH) variables for the different locations varied. In OF conditions, the MIC RH_ave_ was slightly lower compared to the MES in the 2020/2021 season (Table 4). The MIC RH_ave_ of the OPC trial was slightly lower compared to that of the MES in the 2021/2022 and 2022/2023 seasons, except for the W070 and W080 treatments at DS 7, 9, and 10 in 2021/2022, and the W055 treatment at DS 7, 9, and 11 of 2022/2023. In both trials, the respective RH_max_ and RH_min_ trends were as follows: MES RH_max_ > MIC RH_max_ and MIC RH_min_ > MES RH_min_. In the 2020/2021 and 2021/2022 seasons, RH_ave_ and RH_max_ were decreased significantly by W070 compared to W080 from DS 5 towards the end of harvest in the OF trial. In the 2020/2021 season, at DS 5 and in the 2021/2022 season, from DS 7 to 11, the W070 treatment significantly decreased MIC RH_min_ of the OF trial compared to W080 (Table 4 and Table 5). No significant differences were found for RH_min_ in the OF trial in the 2022/2023 season, except for a significant increase that W055 caused compared to W070 and W080 at DS 5 (Table 6). 

Throughout the 2021/2022 and 2022/2023 seasons, W070 caused a higher RH_ave_ underneath the OPC than under OF conditions (Table 5 and Table 6). In the 2022/2023 seasons, except for DS 5, W055 also increased RH_ave_ under OPC compared to OF. Throughout the 2021/2022 season, the W055 treatment underneath OPC decreased all humidity variables compared to the same treatment in the OF trial. From DS 7 to DS 11 of the 2022/2023 season, RH_min_ of the W055 treatment underneath OPC was increased compared to the same treatment under OF conditions (Table 6).

Total radiation of the MES exceeded that of the macro-environment in the 2022/2023 season, whereas an opposite trend occurred in the 2021/2022 season (Table 5 and Table 6). The trend of the differences between the MES- and MAC-radiation in the 2021/2022 and in the 2020/2021 seasons was similar, except for the DS 7 in 2020/2021 (Table 4 and Table 5).

Total radiation values in the MIC of the OF and OPC trials were lower compared to those of the MES and MAC. Underneath the OPC, the total radiation was less than in the OF trial (Table 5 and Table 6). In the OF trial in the 2021/2022 and 2022/2023 seasons, the W070 treatment increased total radiation as well as PAR compared to W100. Photosynthetic active radiation was increased by W055 compared to W100 and W080 at DS 7, 9, and 10 in 2021/2022 in the OF trial. In the OPC trial, PAR was below 30 µmol/m^2^/s for W100, W080 and W070 as well as for W055 at DS 9 and 11 in the 2021/2022 season and DS 5 in the 2022/2023 season.

The transmittance properties of the plastic product changed over time (Table 7). From 2021/2022 to 2022/2023, the direct proportions of transmitted light in the respective wavelength ranges decreased. The diffused components increased in all the wavelength ranges, except the Ultraviolet-B (UV-B) range, which decreased. Reflectance properties for solar radiation, PAR and visible light slightly increased from 2021/2022 to 2022/2023, whereas reflectance of the light in the UV and infrared ranges slightly decreased from one season to the next.

### 2.4. Physiological Variables

#### 2.4.1. Light Intensity

**Figure 3:**
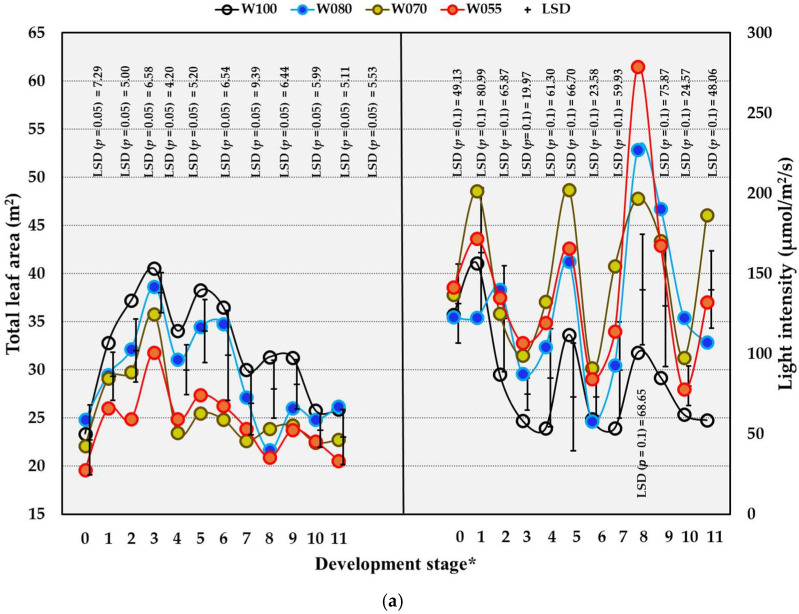

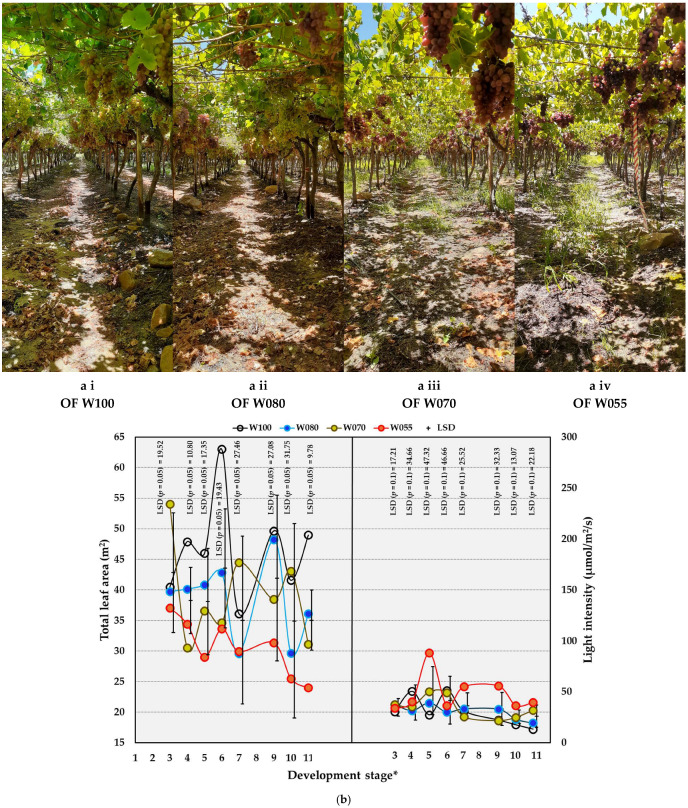

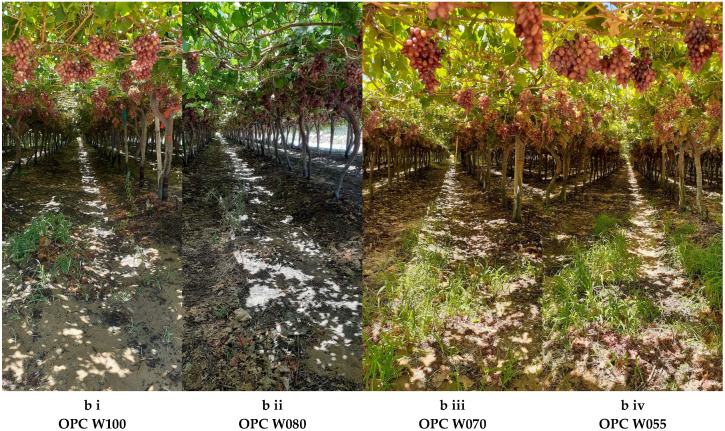
The effect of water (W) treatments on leaf area and light intensity in a Crimson Seedless vineyard grown under open field (OF) conditions from 2019/2020 to 2022/2023 (**a**) and overhead plastic covering conditions (OPC) from 2021/2022 to 2022/2023 (**b**) near Robertson, South Africa. * Development stages: 0 = full bloom, 1 = berry set, 2 = 6 to 11 DAFB (Days after full bloom), 3 = 20 to 25 DAFB, 4 = 34 to 39 DAFB, 5 = 48 to 53 DAFB, 6 = 62 DAFB, 7 = 67 to 77 DAFB, 8 = 78 to 81 DAFB, 9 = 83 to 88 DAFB, 10 = 90 to 95 DAFB, 11 = 97 to 102. W100 (100% = commercial practice), W080 (80% of commercial practice), W070 (70% of commercial practice), W055 (55% of commercial practice). LSD = Least significant difference.

Except for DS 5 and DS 10, W055 significantly increased light intensity compared to W100 in the OF trial (Figure 3a). Reducing the water supply from DS 9 to 11 to 80% also increased light intensity significantly in OF conditions (Figure 3a). Decreasing the water supply underneath OPC had similar effects on light intensity as in the OF trial. Under OPC, the W055 treatment increased the light intensity in the bunch zone from DS 9 to 11 (Figure 3b). The correlations between leaf area and light intensity were moderately negative in both trials, and Pearson correlation coefficients (PCCs) were −0.382 for the OF trial and −0.471 for the OPC trial. These inverse correlations between leaf area and light intensity are demonstrated in Figure 3a(i–iv) for the OF trial and in Figure 3b(i–iv) for the OPC trial. The negative correlations between light intensity and SWC were very weak in the OF trial with a PCC (Pearson correlation coefficient) = −0.117, but very strong in the OPC trial with a PCC = −0.685. The sun fleck patterns on the soil surface indicated how light intensity increased with decreased amounts of water applied in both trials (Figure 3). The amount of sunlight reaching the soil surface was more in the OF trial compared to that in the OPC trial, the latter only being 12–30% of the light under OF conditions.

The effects of the water treatments on the total leaf area were more pronounced in the OF trial as opposed to the OPC trial (Figure 3). The decreases in total leaf area caused by all the treatments compared to W100 in the OF trial were gradual (Figure 3a). Except for the gradual trend of W055 over time, the responses due to decreased water supply in the OPC trial were erratic, with large fluctuations between development stages (Figure 3b). Compared to W100, the W055 and W070 treatments caused significant decreases in total leaf areas from DS 2 to 6, at DS 8, and at DS 9. At DS 8 and 9, W080 also decreased total leaf area significantly compared to W100. At DS 4, total leaf area was decreased significantly by W055 and W070 compared to W100 in the OPC trial. At DS 6 and at DS 11, all the treatments significantly decreased total leaf area compared to W100 underneath OPC. The responses of leaf area to reduced water supply treatments were ascribed to SWC. There were relatively strong correlations between SWC and total leaf area per vine in the OF trial (PCC = 0.58) and the OPC trial (PCC = 0.574). 

Seasonal differences for light intensity and leaf area did not follow clear trends in the OF and OPC trials (Table 8). 

#### 2.4.2. Stem Water Potential

Season and treatment interactions were found for stem water potential (Ψ_S_) from DS 9 to 11 in the OF trial (Table S1). These interactions only occurred at DS 3 and 11 in the OPC trial, as indicated by the probability values of the F-ratio test. 

**Figure 4:**
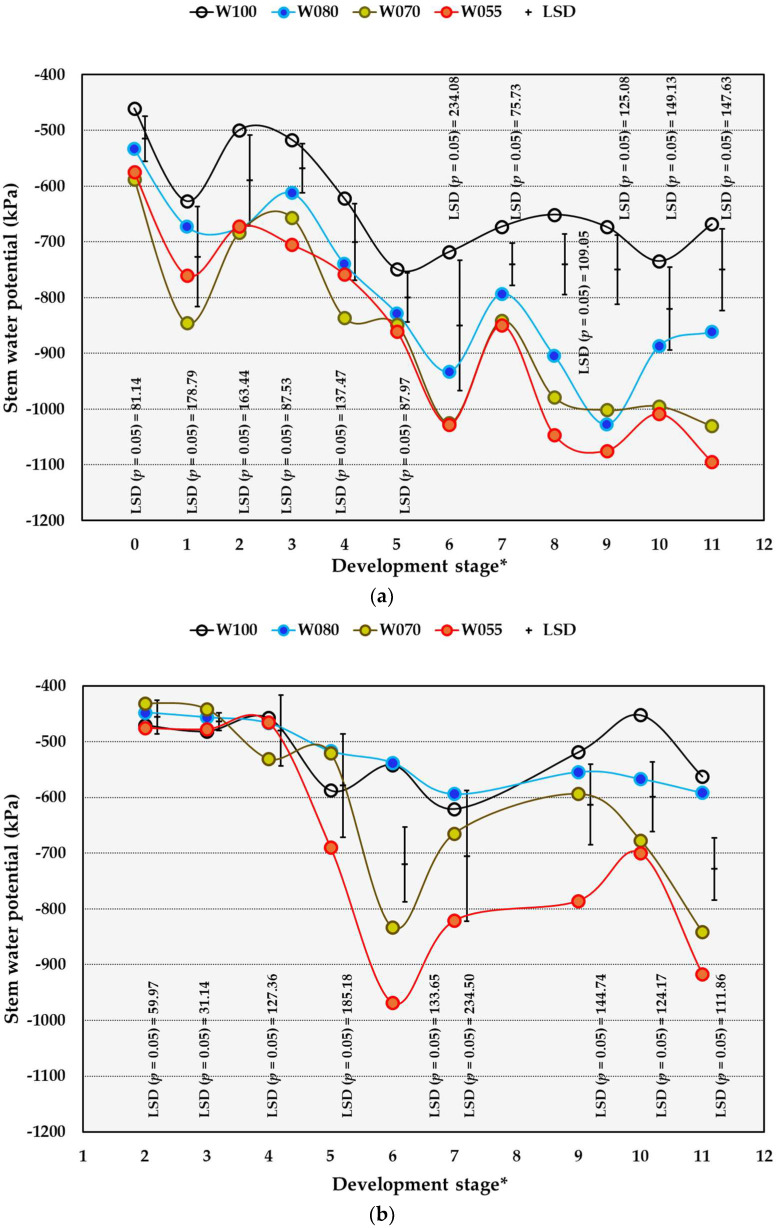
The effect of water (W) treatments on the stem water potential of Crimson Seedless grown under open field conditions from 2019/2020 to 2022/2023 (**a**) and overhead plastic covering conditions from 2021/2022 to 2022/2023 (**b**) near Robertson, South Africa. * Development stages: 0 = full bloom, 1 = berry set, 2 = 6 to 11 DAFB (Days after full bloom), 3 = 20 to 25 DAFB, 4 = 34 to 39 DAFB, 5 = 48 to 53 DAFB, 6 = 62 DAFB, 7 = 67 to 77 DAFB, 8 = 78 to 81 DAFB, 9 = 83 to 88 DAFB, 10 = 90 to 95 DAFB, 11 = 97 to 102. W100 (100% = commercial practice), W080 (80% of commercial practice), W070 (70% of commercial practice), W055 (55% of commercial practice). LSD = Least significant difference.

From DS 2 to 5 and at DS 7, the Ψ_S_ of W070 and W055 in the OF trial was significantly lower compared to the Ψ_S_ of W100. Later in the season, from DS 8 to 11, W055 and W070 in the OF trial resulted in Ψ_S_ values significantly lower than the Ψ_S_ of W100. The W070 treatment decreased Ψ_S_ significantly compared to W100 in the OF trial throughout the growth and ripening period (Figure 4a). In comparison to the control, W055 also caused a continuous significant decrease in Ψ_S_ under OF conditions, except for DS 2 and 4. Compared to W100, the W080 treatment decreased Ψ_S_ significantly from DS 9 to 11 in the OF trial. From DS 9 to 11, W055 decreased Ψ_S_ significantly compared to W100 underneath OPC (Figure 4b). The W070 and W055 treatments decreased Ψ_S_ significantly at DS 3, and again at DS 6, as well as at DS 10 and 11 underneath OPC. In the 2022/2023 season, Ψ_S_ from DS 9 to 11 was significantly higher compared to all preceding seasons in both trials (Table 9). Strong positive correlations between average SWC and Ψ_S_ [(PCC = 0.726 (OF); PCC = 0.820 (OPC)] as well as between the total leaf area and Ψ_S_ [(PCC = 0.573 (OF); PCC = 0.574 (OPC)] were found. 

#### 2.4.3. Photosynthesis and Transpiration

**Figure 5:**
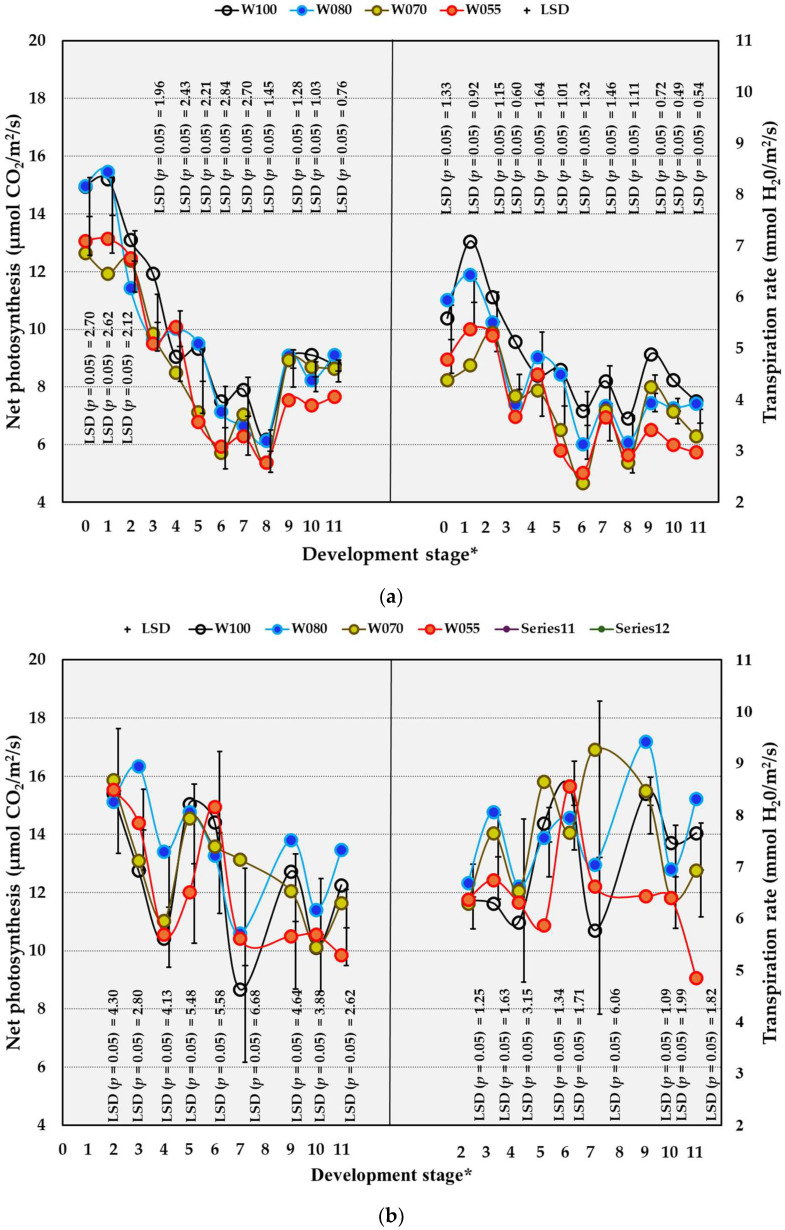
The effect of water (W) treatments on photosynthetic and transpiration rates of Crimson Seedless grown under open field conditions from 2019/2020 to 2022/2023 (**a**) and overhead plastic covering conditions from 2021/2022 to 2022/2023 (**b**) near Robertson, South Africa. * Development stages: 0 = full bloom, 1 = berry set, 2 = 6 to 11 DAFB (Days after full bloom), 3 = 20 to 25 DAFB, 4 = 34 to 39 DAFB, 5 = 48 to 53 DAFB, 6 = 62 DAFB, 7 = 67 to 77 DAFB, 8 = 78 to 81 DAFB, 9 = 83 to 88 DAFB, 10 = 90 to 95 DAFB, 11 = 97 to 102. W100 (100% = commercial practice), W080 (80% of commercial practice), W070 (70% of commercial practice), W055 (55% of commercial practice). LSD = Least significant difference.

In the OF trial, treatment and season interaction for net photosynthesis (Pn) and transpiration (Tr) occurred only at DS 6 and DS 9, whereas no treatment and season interaction occurred underneath the OPC (Table S1). In the OF trial, the W055 treatment (compared to W100) decreased Pn and Tr rates significantly at DS 3 and DS 5, as well as from DS 9 to 11 (Figure 5a). Compared to W100, the W070 and W080 treatments significantly decreased the Pn and Tr rates only at DS 3. The general lack of significant differences between W100 and W080 for photosynthesis indicated that W080 was sufficient for photosynthetic performance. Underneath the OPC, Pn and Tr increased significantly by W080 compared to W100 at DS 3 (Figure 5b). At DS 5, 9, and 11, Tr decreased significantly by W055 compared to W100 in the OPC trial. Towards the end of the season, Pn reached a much lower rate in the OF trial compared to the OPC. Underneath the OPC, the effect of the water treatments on Pn was not as pronounced as in the OF trial. Transpiration followed Pn under OF conditions. However, underneath the OPC, transpiration remained stable for all treatments, except for W055. Underneath the plastic, the Pn and Tr rates were higher compared to OF conditions.

Considering the seasonal differences in the OF trial, the Pn and Tr rates were significantly higher from DS 9 to 11 in the 2022/2023 season compared to all the previous seasons (Table 10). Under OPC, the Pn rates were significantly higher in the 2022/2023 season compared to the 2021/2022 season only at DS 10, whereas Tr rates in 2022/2023 were higher only at DS 3 and 4 (Table 10). The PCCs showed strong positive correlations between SWC and Pn in the OF trial (PCC = 0.596) as well as in the OPC trial (PCC = 0.575). The correlation between SWC and Tr was strongly positive (PCC = 0.634) only in the OF trial. 

#### 2.4.4. Photosynthesis: Transpiration Ratio

**Figure 6:**
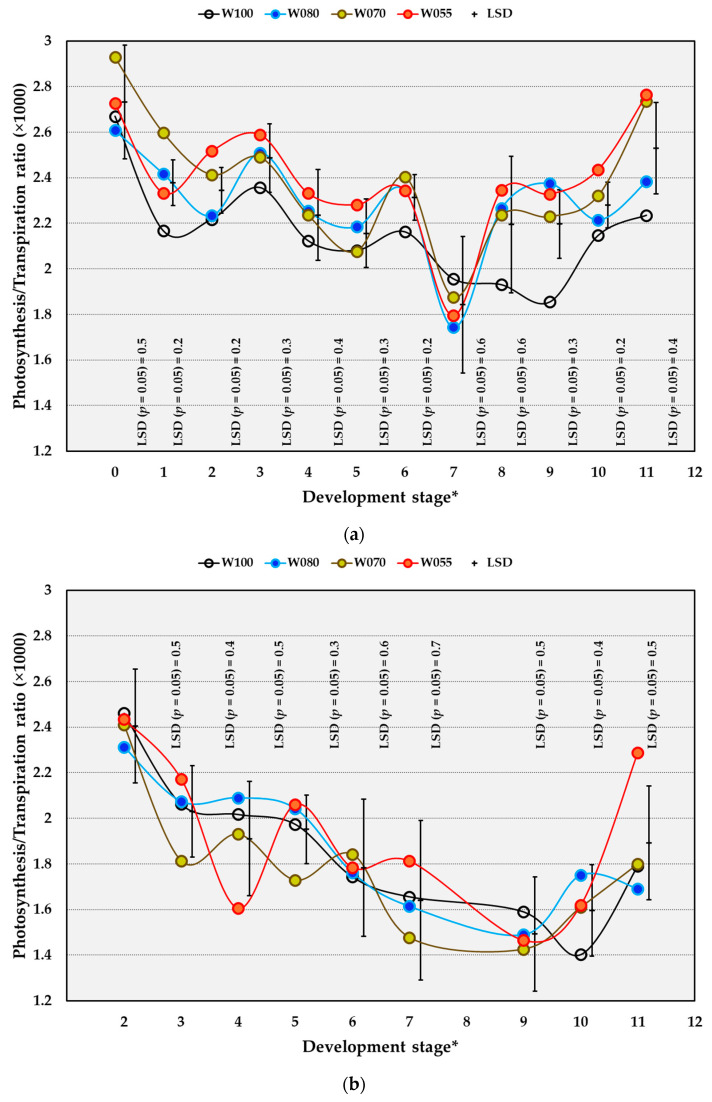
The effect of water (W) treatments on photosynthesis/transpiration ratio of Crimson Seedless grown under open field (**a**) and overhead plastic covering (**b**) conditions near Robertson, South Africa, for the 2019/2020 to 2022/2023 seasons. * Development stages: 0 = full bloom, 1 = berry set, 2 = 6 to 11 DAFB (Days after full bloom), 3 = 20 to 25 DAFB, 4 = 34 to 39 DAFB, 5 = 48 to 53 DAFB, 6 = 62 DAFB, 7 = 67 to 77 DAFB, 8 = 78 to 81 DAFB, 9 = 83 to 88 DAFB, 10 = 90 to 95 DAFB, 11 = 97 to 102. W100 (100% = commercial practice), W080 (80% of commercial practice), W070 (70% of commercial practice), W055 (55% of commercial practice). LSD = Least significant difference.

The Pn/Tr ratio in the OF trial showed the most prominent responses from DS 9 to 11, especially for vines receiving W055 (Figure 6a). Compared to the W100 treatment, W055 increased the Pn/Tr ratio significantly from DS 9 to 11, and W070 caused a significant increase in Pn/Tr at DS 6, 9, and 11 in the OF trial (Figure 6a). During the berry growth phase, W070 significantly increased the Pn/Tr ratio only at DS 1, whereas W055 increased the Pn/Tr ratio at DS 2 in comparison to W100 in OF conditions. Compared to the OF conditions, the Pn/Tr ratio underneath the OPC was considerably lower. Compared to W100, none of the treatments had a significant effect underneath OPC (Figure 6b). In the 2020/2021 season, the Pn/Tr ratio was higher than in all the other seasons at DS 5, 7, and DS 8–10 in the OF trial (Table 10). In the OPC trial, the Pn/Tr ratio was highest in the 2022/2023 season at DS 2, 4, and 10. Underneath the OPC, the photosynthetic water use efficiency (WUE) was lower compared to the WUE in the OF trial, as indicated by the lower Pn/Tr ratio obtained underneath the OPC. Underneath OPC, increased Tr caused a lower WUE.

## 3. Discussion

The direct relationship between clay contents and soil water content [39] were applicable to the differences in WHC. The differences in WHC between the W070 and W080 treatment plots of the OF trial were ascribed to texture differences, i.e., higher clay content and lower sand content of the W070 treatments (Table 1). 

Calcium and Mg dominated the exchange complex of the clay particles in the OF and OPC trials. The cation exchange capacity of clay particles was evident at 30 cm soil depth in the W070 and W080 sampling plots. The higher clay content in the sampling plots of the W070 treatments may explain the lower base saturation values for Ca and the higher base saturation values for Mg in the sampled plots of the W070 treatments as opposed to the sampled plots of the W080 treatments in the OF trial [40]. Increased K in the W070 plots compared to the W080 plots was likely due to the release of K from the clay particles, which could have been supported by the high WHC of the W070 treatment [41]. Water holding capacity, the base saturation values for Ca and Mg, as well as the exchangeable K for the W070 as opposed to the W080 sampling plots, were all related to the clay content.

The pH_KCl_ values ranging between 5.5 and 6.5 were within the ideal range for grapevines [39]. With the pH_KCl_ values within the acceptable range, the availability of essential nutrients, such as Ca and Mg, was thus ensured [39].

The W070 treatment resulted in similar SWC to that of the W080 treatment, despite higher volumes of water applied in the latter. As reported by Ramos et al. [42], soils with a higher WHC, such as the soil in the W070 treatment plots of the OF trial (Table 1), maintain more moisture for longer. Similarly, Martínez-Vidaurre et al. [43] reported that soils with a higher WHC had more available water, resulting in Tempranillo/Richter 110 vines being less stressed. The results of Tramontini et al. [3] demonstrated the importance of soil water availability for the improvement of rates of CO_2_ assimilation, transpiration, and stomatal conductance. The higher SWC in the OPC trial may be ascribed to the covers providing shade. Overhead plastic covers lower evapotranspiration by decreasing the radiation reaching the soil surface, lowering evaporation [21,24,25].

Average air T in the MIC never increased to above 35 °C (Table 4, Table 5 and Table 6), the temperature above which net photosynthesis is known to decline [6]. The air RH considered optimal for photosynthetic activity of grapevines is 60% to 70% [35]. In this study, RH_ave_ in both trials was well within this range. Considering previous results [6,33], temperatures in the MIC of the OF and OPC trials were suitable for optimal photosynthesis and vine performance. 

The increased air T variables underneath the OPC from the 2021/2022 season to the 2022/2023 season are ascribed to product degradation, leading to increased transmittance of radiation, especially diffused solar infrared light [23]. Although the product degraded from one season to the next, the decrease in temperatures underneath the OPC compared to the OF in the 2021/2022 season could be an advantage under higher ambient temperature conditions. Global surface temperatures are predicted to increase between 1.5 °C and 4 °C by the year 2100 [44], with implications for grape-growing areas worldwide due to the impact on grapevine development as well as grape quality [45,46,47]. Covering table grape vineyards in areas where temperatures are increasing would thus mitigate climate change.

The total radiation of the MES (of the vineyards on the farm) exceeding that of the MAC (larger environment) in 2022/2023 was likely due to an increased surface albedo [48], ascribed to newly established buildings and vineyards covered with shade nets on the farm, increasing the reflective portion of radiation [49]. 

The increase in diffusivity of the OPC from 2021/2022 to 2022/2023 in all the wavelength ranges, except the UV-B range (Table 7), was ascribed to plastic product degradation.Plastic product degradation is one of the factors that most likely impacted photosynthesis and the thermal properties of the leaves and the bunch zones in the OPC trial in the 2022/2023 season. The decrease in diffused UV-B over time held possible implications for phenolic content because solar UV-B stimulates the synthesis of, amongst others, phenols in leaves and/or grape berries [28,50]. The increase in the diffused component of light underneath the OPC increased the ability of light to penetrate the gaps in the canopy [23,51]. Thus, the increased diffuse component could have improved the photosynthetic efficiency of the leaves in the OPC trial. According to Smart [52], leaves that also receive diffused light had higher photosynthetic efficiency, although approximately 70% of photosynthesis is ascribed to direct light. Furthermore, diffused light is more beneficial in terms of, amongst others, berry temperature and sunburn control [53]. Therefore, the use of OPC could decrease the risk of sunburn, especially for cultivars prone to sunburn.

The reduced reflection of the OPC in the 2022/2023 season and thus the partial retention of the long infrared radiation underneath the covers could account for the tendency of increased temperatures underneath the OPC compared to OF conditions [23].

The lower radiation values obtained in the OPC trial were ascribed to the shielding effect of the covers. Underneath OPC, the light intensities ranged between 13 and 88 µmol/m^2^/s, thus within the light compensation range of between 10 and 30 µmol/m^2^/s [33,54,55]. However, the possible negative effects of such low light intensity values were probably counteracted by the increased diffused component of light underneath the OPC [52]. Therefore, the benefits of increased diffused transmittance might be an advantage in conditions where light intensity in the micro-climatic environment needs to be increased.

The overall strong correlations between SWC and total leaf area per vine showed the dependence of leaf area development on water. Similarly, Pellegrino et al. [56] reported significantly lower leaf areas for pot-grown Shiraz grafted onto Fercal rootstock subjected to severe water deficits compared to the control treatment in a greenhouse. The strong negative correlation between light intensity and SWC in the OPC trial confirmed the correlations that are generally found between canopy size and light intensity [57,58]. Stem water potential is used as an indicator for the degree of water deficit experienced by grapevines in wine and table grape cultivation. Table grape quality depends on sufficient water supply to maintain turgor [59]. Water status thresholds, indicated by Ψ_S_, showed deficits ranging from mild or weak to severe at higher values (lower negative) for table grapes than for wine grapes [13,38]. Therefore, narrower Ψ_S_ ranges for the water status thresholds are more applicable to table grapes.

Based on the summarised Ψ_S_ values of Rienth and Scholasch [13], no water deficiency was detected from DS 0 to 3 and only a mild deficit from DS 3 to 11 for the W100 treatment in the OF trial (Figure 4a). The W080, W070, and W055 treatments showed no water deficit at DS 0, a mild deficit from DS 1 to 5, and fluctuations between mild and moderate from DS 6 to 11. While in the OF trial water deficits of the W100 treatment increased to a mild level at DS 4, the W100 treatment underneath the OPC mostly remained in the no deficit zone for the duration of the trial. Except for W100 at DS 7, the W100 and W080 treatments showed no deficit throughout the development stages underneath the OPC (Figure 4b). The W070 treatment underneath OPC caused no deficit from DS 2 to 5, and at DS 9, and only mild deficits at DS 6, 7, 10, and 11 (Figure 4b). Mild deficits occurred at DS 5 and DS 7–10, and moderate deficits at DS 6 and 11 underneath OPC due to W055, compared to W100. 

Based on the threshold Ψ_S_ values for table grapes [38], none of the treatments indicated a deficit at DS 0 in the OF trial (Figure 4a). At DS 2 and 3, the values for the W100 treatment fell in the no deficit zone and moved to a weak deficit zone from DS 4 to 11 in OF conditions. The W080 treatment in the OF trial caused a weak deficit from DS 1 to 4, and from DS 5 towards DS 11 fluctuated between moderate and weak, with a strong deficit at DS 9. The W070 and W055 treatments in the OF trial fluctuated between weak and moderate between DS 1 and DS 5, whereas fluctuations between moderate and strong occurred after DS 5. Underneath OPC, the Ψ_S_ values for the W100 treatment were in the no deficit zone at all DS, except DS 7 (Figure 4b). The Ψ_S_ values for W080 were consistently in the no deficit zone in the OPC trial. Vines underneath OPC and subjected to W070 experienced no deficit from DS 2 to 5, whereas deficits fluctuated between weak and moderate from DS 6 to 11. The W055 treatment applied underneath OPC resulted in Ψ_S_ values corresponding to no deficit from DS 2 to 4 and fluctuating between weak and moderate from DS 5 to 11.

From DS 2 to 5 and at DS 7, the Ψ_S_ of W070 and W055 in the OF trial corresponded to mild degrees of water deficit. Later in the season, from DS 8 to 11, W055 and W070 in the OF trial resulted in Ψ_S_ values that corresponded to moderate deficits. At DS 9 in the OF trial, the Ψ_S_ value caused by W080 corresponded to a moderate level of water deficit, and at DS 10–11, the Ψ_S_ values improved and indicated mild deficit levels.

Underneath the OPC, the Ψ_S_ values were higher compared to those in the OF trial, thus showing that the Crimson Seedless vines were less stressed. While in the OF trial water deficits of the W100 treatment increased to a mild level at DS 4, the W100 treatment underneath the OPC mostly remained in the no deficit zone for the duration of the trial. Furthermore, the W070 and W055 treatments showed higher values for Ψ_S_ underneath the OPC than in the OF trial. The W070 treatment underneath OPC caused no deficit from DS 2 to 5, and at DS 9, and only mild deficits at DS 6, 7, 10, and 11. Mild deficits at DS 5 and DS 7–10 and moderate deficits at DS 6 and 11 underneath OPC due to W055 compared to W100 occurred. Except for W100 at DS 7, the W100 and W080 treatments showed no deficit throughout the development stages underneath the OPC.

The strong positive correlations between SWC and Ψ_S,_ as well as between the total leaf area and Ψ_S,_ showed how grapevines can adjust their water use to soil water availability [2]. In line with previous findings, the soil water status reflected in the vines showed that soil water availability is the most important for assimilation rate, transpiration rate, and stomatal conductance [1,3,38]. The correlation between leaf area and stem water potential indicated that a higher leaf area reduced the amount of radiation reaching the soil surface, thus preserving the SWC [25]. In both trials, Ψ_S_ integrated the effects of the soil, plant, and atmospheric conditions and thus gave a good indication of grapevine water status [13,38]. Furthermore, assimilation rate, transpiration rate, and stomatal conductance are the most important indicators of water availability in the soil. The improved photosynthesis and transpiration rates underneath OPC were ascribed to the higher soil water content, as emphasised previously [1,3,38]. In the OPC trial, the soil water content was conserved due to shaded conditions and less evapotranspiration [24,25]. Water availability can mitigate the negative effects of high temperatures on photosynthesis by increasing transpiration rates, leading to evaporative cooling [8,10,60]. Higher photosynthetic rates equated with open stomata, hence increased transpiration. The lower Pn/Tr ratio obtained underneath the OPC indicated a lower photosynthetic WUE compared to the OF. As opposed to vines that were well supplied by water, the vines under deficit conditions in both trials responded physiologically by decreasing Pn and Tr.

## 4. Materials and Methods

### 4.1. Vineyard and Plant Material

The trials were conducted on 11-year-old Crimson Seedless (*Vitis vinifera* L.) grapevines, grafted onto Ramsey (*Vitis champinii*). Grapevines of the Crimson Seedless cultivar belong to the *Vitaceae* family and are classified into the *Vitis* genus and the *vinifera* species [61,62,63]. Crimson Seedless, a red variety, was developed in California in 1979 and is the result of a Fresno C33-199 x Emperor cross [64]. Typically, the bunches are medium-sized (+−0.5 kg) with a conical shape [64]. The cultivar was imported to South Africa by the Institute for deciduous fruit, vines and wine (currently known as the Agricultural Research Council Infruitec-Nietvoorbij, Stellenbosch) in 1990 [65]. After propagation by Plant Improvement South Africa (PlantSA), Crimson Seedless vines were distributed to various locations in different cultivation areas and the first commercial plantings in South Africa were established in 1996 [65]. Plant material for the vineyard used for the trials in this study was obtained from a commercial nursery in 2008. 

The approximate stem height of the grapevines was 1.5 m from the soil surface to the top of the vines where the bearing shoots emerged. An open field (OF) trial and a trial underneath overhead plastic covering (OPC) were conducted in a commercial vineyard in the Breede River Valley of South Africa (33°47′24.87″ S, 19°41′4.24″ E). Treatments were applied and data collected over four seasons (2019/2020 until 2022/2023) in the OF trial, and over two seasons (2021/2022 and 2022/2023) in the OPC trial. Vines were trained onto a pergola trellis system and were spaced 1.75 m × 3.0 m on a stony, loam-sand soil. Irrigation took place by means of 360° penned Gulf micro-sprinklers (Agriplas, Cape Town, WC, South Africa) with a 32 L/h delivery rate and 1.6 m spacing straight below the vines on the soil surface. 

Standard viticultural practices were applied according to the guidelines prescribed by the South African Table Grape Industry [66]. During the winter months, all the experimental vines in both trials were pruned to 10 long bearers (canes) with 12 buds each. To ensure bearers in the next season, a short bearer (spur) with two buds was left at the base of each cane. Non-allocated shoots were removed from old wood before flowering. Shoots were distributed to prevent crowding the canopy. After shoot distribution, the shoots were tied to the canopy wires. After berry set, bunches were shortened to approximately 10 cm from the first lateral branch to the basal bunch tip. Leaves in the bunch zone were removed. To improve berry size, the vine trunks were girdled in the middle of the trunk when the transverse berry diameter reached 4–5 mm. The crop load was reduced to approximately 30 bunches per vine. At transverse berry diameters of 9–10 mm, bunch laterals were removed (berry thinning) to loosen bunches. Colour development was enhanced with 200 ppm ethephon, applied three to five days apart between 5% and 10% véraison. The soil surface inside the vineyard was sown with rye in autumn, and the growth stand was chemically controlled before budbreak. Macro- and micro-element fertilisers applied during the growth season were calculated based on annual soil analyses after the growth season. 

### 4.2. Experimental Design and Treatments

**Figure 7:**
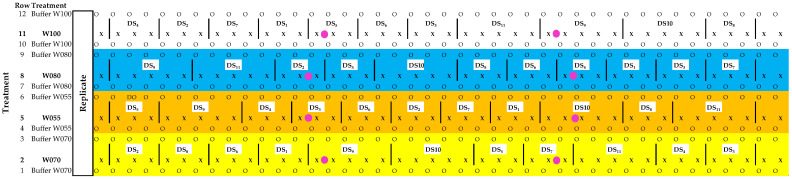
Experimental unit per treatment per replicate in each trial: W = water treatment; W100 = 100% water application; W080 = 80% water application; W070 = 70% water application; W055 = 55% water application; X = treatment vines; O = buffer vines; | = lines indicating group of sampling vines; DS = Development stage sampling vines; DS 1–8 = sampling times before harvest; DS 9–11 = sample times during harvest period. 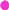 = neutron probe measurement sites.

Four water (W) treatments were applied by means of irrigation, which included W100, W080, W070, and W055, corresponding to 100%, 80%, 70%, and 55%, respectively, of commercial practice. For each micro-climatic condition, the experimental design was a randomised complete block with the four treatments replicated in six blocks each for OF conditions and four blocks each for OPC. Each block replicate of a specific water treatment (experimental unit) consisted of a row of 42 vines. Cross-contamination between the different irrigation treatments was prevented by incorporating buffer rows (Figure 7).

Sampling and harvest dates were expressed as development stages (DS), and were indicated according to the number of days after full bloom (DAFB) from 0 to 11 and the BBCH scheme [67], where 0 = full bloom (BBCH 61), 1 = berry set (BBCH 71), 2 = 6 to 11 DAFB (BBCH 73), 3 = 20 to 25 DAFB (BBCH 75), 4 = 34 to 39 DAFB (BBCH 77), 5 = 48 to 53 DAFB (BBCH 79), 6 = 62 DAFB (BBCH 81), 7 = 67 to 77 DAFB (BBCH 83), 8 = 78 to 81 DAFB (BBCH 85), 9 = 83 to 88 DAFB (Harvest 1; BBCH 89), 10 = 90 to 95 DAFB (Harvest 2; BBCH 89), 11 = 97 to 102 DAFB (Harvest 3; BBCH 89). At each development stage prior to harvest (development stages 0–8), a different group of three randomly selected vines per experimental unit were selected for measurements (Figure 7). At each harvest date (development stages 9–11), sampling was performed from a different group of five randomly selected vines per experimental unit. 

The required irrigation volumes applied for the control treatment (W100) were expressed in terms of crop evapotranspiration (ET_c_). Crop evapotranspiration was calculated using crop coefficients (K_c_) adapted from published values [68,69]. Reference evaporation rates (ET_0_) were computed with data from a nearby weather station, using the equation ET_c_ = K_c_ × ET_0_ [68]. Crop coefficient values ranged from 0.3 to 0.6 from the start of the growth season until harvest. Water treatments were applied in one or two supplies during the week, depending on ambient temperature and evaporation. Irrigation volumes for the treatments were regulated with additional taps installed at the end of each row, between the feeding line and irrigation line. In four replicates of each treatment in the OF trial (n = 16) and the OPC trial (n = 16), water meters were installed to monitor irrigation volumes.

**Figure 8:**
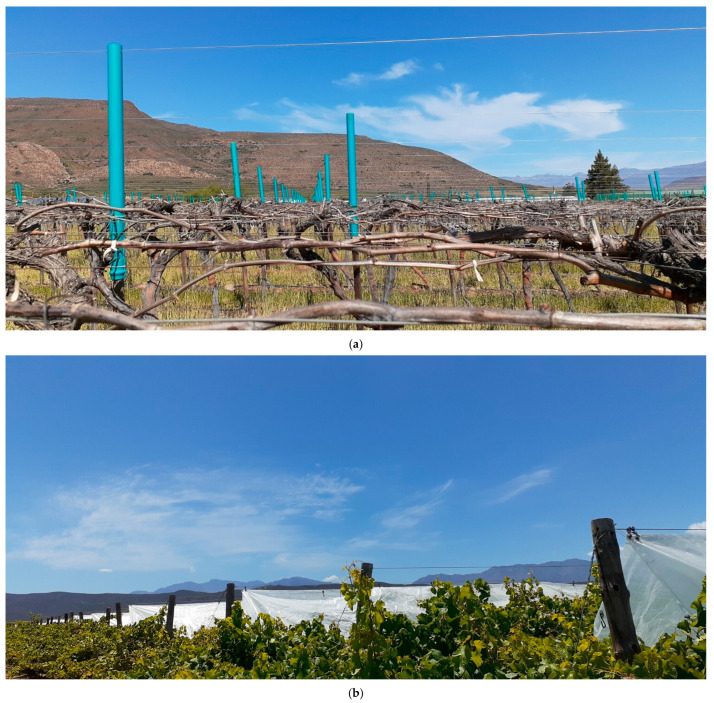
Overhead plastic cover installation showing the adapted pergola trellis system with polyvinyl chloride pole extensions and overhead cables (**a**), and the installed covers (**b**).

The micro-climatic environment in the vineyard was altered with lightweight woven overhead plastic covers consisting of ±93% polypropylene, ±6% polyethylene, and <1% UV stabiliser (Polypropylene Products, Durban, KZN, South Africa). To enable installation of the covers above the canopy, the existing pergola trellis system was modified by extending the poles by 1.1 m with polyvinyl chloride tubes that were fitted over the top of the existing wooden poles (Figure 8a). Overhead cables were strung through holes at the top of each extension tube at a height of 1.095 m above the top of the existing trellis poles. To fit the specified in-row distances between the extended poles, the plastic covers were manufactured as 3.2 m wide strips of different lengths. The plastic strips were installed from the overhead cable above the centre of each row at equal distances sideways towards an additional steel wire, installed at the same height as the canopy wires, to form a triangular shape (Figure 8b). The covers were attached to the overhead cables and the additionally installed steel wires by means of S-shaped hooks. To ensure continuous coverage of each row, the individual plastic cover strips between the extended poles were connected to each other with S-shaped hooks and steel wire. The distance between the top of the vines and the top of the plastic covers was approximately 1.0 m. The OPC was installed after budbreak between BBCH 05 and 19 [19], and the sides of the vineyard remained open. The covers were installed in November 2021 and removed in May 2022. They were re-installed in the following season in November 2022 and removed in May 2023. 

Before installation of the covers (2021), and after completion of the trial (2023), the transmittance and reflectance properties of the plastic covers were determined on three randomly sampled product material sheets with dimensions 210 × 297 mm. Radiometric properties of the plastic were determined as described by [70]. Spectral transmissivity and reflectivity measurements within the 200–2500 nm wavelength range were carried out with a double beam UV-VIS-NIR Lambda 950 spectrophotometer (Perkin Elmer Instruments, Norwalk, CT, USA) and a 60 mm diameter integrating sphere. Within the 2500–25,000 wavelength range, transmissivity was measured with an FT-IR 1760 X spectrophotometer (Perkin-Elmer Instruments, Norwalk, CT, USA). Transmissivity was measured using radiation with direct perpendicular incidence, whereas reflectivity was measured at near-normal incidence (10°). Transmissivity and reflectivity coefficients were calculated with the equations described by Vox and Schettini [70]. Radiometric properties of the plastic product are shown in Table 7.

### 4.3. Water Application Volumes and Soil Water Measurements 

To determine SWC, two measurement sites per treatment row per trial were prepared when the trials were established (Figure 7). The measurement sites were prepared by drilling holes to a depth of 100 cm with a soil auger without disturbing the soil adjacent to the hole. Each hole was lined with a tight-fitting 90 mm PVC class 9 tube with a sealed bottom end. The top ends of the tubes were equipped with removable covers to prevent irrigation and rainwater from filling the tubes. The soil water content was measured with a Hydroprobe™, (CPN, Martinez, CA, USA). Soil count: air count ratios, with the neutron probe in the storage position, were calibrated against gravimetric soil water content at increments of 30 cm, starting at 30 cm, up to a depth of 90 cm. The gravimetric method entailed weighing a moist sample of soil taken at 30 cm, 60 cm, and 90 cm of all the treatment plots of one replicate (n = 36) in each trial. The samples were oven-dried at 100 °C for 72 h and reweighed. The mass of water lost per sample was calculated as a percentage of the mass of the dried soil.

Linear regression between the gravimetric SWC and the probe count ratio was used to calculate calibration curves. Probe measurements were taken at each probe tube at 30 cm depth intervals, from 30 cm to 90 cm. Count measurements started before the application of the first irrigation treatments at the end of October and thereafter bi-weekly, one day before irrigation during the sampling period and two days before irrigation during the harvest period. 

### 4.4. Soil Analyses

At the end of the harvest season in 2019, soil samples were collected randomly in each treatment row per replicate from 0 to 30 cm and 30 to 60 cm depths. The soil samples of the two trials were analysed by a commercial laboratory (Bemlab Pty Ltd., Cape Town, South Africa) accredited by the South African National Accreditation System (SANAS) in accordance with recognised international standards [71]. Soil texture, pH, macro- and micro-elements, resistance, basic cation saturation, as well as water holding capacity were determined by the respective methods referred to in the facility accreditation schedule [72].

### 4.5. Climatic Measurements

The macro-climatic data was collected from 2019/2020 to 2022/2023 and included mean monthly temperature, rainfall, and radiation. The data was recorded with a weather station of the Agricultural Research Council Institute for Soil, Climate and Water [73] located at 33°46′30.92′′ S, 19°40′13.65′′ E, 2.12 km from the trial site. An on-farm weather station [74] was positioned 323 m from the trial at 33°47′32.30′′ S, 19°41′9.34′′ E, and provided the meso-climatic data around the vineyard on the farm. Inside the vineyard, in the OF and OPC trials (33°47′24.87′′ S, 19°41′4.24′′ E), micro-climatic variables were recorded.

Vaisala HMP 50/60 sensors (Vaisala, Helsinki, Finland), installed underneath the plastic covering and in open field conditions at a height of approximately 1.8 m above the soil surface, measured temperature (°C) and relative humidity (RH). Li-Cor-200 R pyranometers and Li-190 R quantum sensors (Li-Cor^®^ Biosciences, Lincoln, NE, USA) were positioned horizontally in the middle of the vine, 30 cm below the bunch zone, to measure global (direct plus diffuse) radiation in the infrared spectrum of 700–1100 nm, PAR in the 400–700 nm spectrum, respectively. A group of sensors was placed at each of four water treatments in the OF trial and each of four water treatments in the OPC trial. 

### 4.6. Leaf Area Measurements

Shoot sampling took place bi-weekly during principal growth stages seven and eight, at the various growth phases during BBCH 71–BBCH 79 and BBCH 81–BBCH 89 [67]. The development stages (DS) within these growth phases were indicated according to the number of days after full bloom (DAFB) from 0 to 11, as described in Section 4.2 (Experimental design and treatments). Five shoots per treatment per replicate were sampled randomly from each experimental unit. At each development stage, the number of main and lateral shoot leaves was counted, and leaf areas were measured with a LI 3100 area metre (Li-Cor^®^ Biosciences, Lincoln, NE, USA). Total leaf area per vine was calculated by multiplying the leaf area per shoot by the estimated number of shoots per vine, calculated based on the number of shoots and the number of nodes per shoot allocated during pruning.

### 4.7. Physiological Measurements

Measurement of all the physiological variables took place bi-weekly, one day prior to irrigation in the growth season, and one day prior to harvest/two days prior to irrigation, during the harvest period. The measurements started at berry set and continued until the third harvest date. Measurements of all the physiological variables took place from 10:30 until mid-day at the development stages described in Section 4.2 (Experimental design and treatments).

Photosynthetic active radiation (PAR; 400–700 nm) readings were taken in the middle of the bunch zone, parallel to the cordons on both sides of the vine row, approximately 30 cm below the bunches with a Li-Cor Li-190 line quantum sensor (Li-Cor^®^ Biosciences, Lincoln, NE, USA) (µmol/m^2^/s). Six readings in each of three replicates were taken per experimental unit per treatment in the OF trial (n = 72) and in the OPC trial (n = 72) between 10:30 and 12:00. At the start and at the end of the measurement period, readings were also taken outside of the vineyard to record the ambient light intensity. 

Stem water potential (Ψ_S_) measurements (−kPa) were performed on primary shoot leaves in the bunch zone for all the treatments in each of three replicates in the OF trial and in the OPC trial. Two leaves per treatment per replicate of three replicates in the OF trial (n = 24) and two leaves per treatment per replicate of three replicates in the OPC trial (n = 24) were covered with plastic bags lined with aluminium foil for at least one hour prior to measurement. Stem water potential measurements were performed with two Scholander pressure chambers [75] that were equally calibrated at a flow rate of 1000 kPa/30 s and operated by two trained operators. The intensities of water stress were classified according to published values [13,38] as summarised in Table 11.

Photosynthetic rate (µmol CO_2_/m^2^/s), transpiration rate (mmol/m^2^/s), relative humidity (%), air temperature (°C) and photosynthetic photon fluence rate (μmol/m^2^/s) measurements were taken on intact primary shoot leaves from the outer layer of the canopy in the OF trial and in a similar position just below the plastic in the OPC trial. Three primary shoot leaves per treatment per replicate in three replicates in the OF trial (n = 36) and three primary shoot leaves per treatment per replicate in three replicates in the OPC trial (n = 36) were measured. Measurements of photosynthetic and related variables were performed with an open system Li-Cor LI-6400 portable photosynthesis meter (Li-Cor^®^ Biosciences, Lincoln, NE, USA) with gas flow adjusted to 400 ppm CO_2_ and the Li-Cor 6400-02B red/blue light source activated at 2000 µmol/m^2^/s. 

### 4.8. Statistical Analyses

All measured variables were subjected to analysis of variance (ANOVA) according to the randomised block experimental design, for each trial, season, and development stage separately. The season was included as a repeated measures factor per trial and development stage. For all variables, treatments were compared within each climate and season as well as per climate with seasons as repeated measures factor using the general linear model (GLM) procedure [76]. The Shapiro–Wilk test was performed to test for deviation from normality [77]. Outliers were removed when the standardised residual for an observation deviated with more than three standard deviations from the model value. For all means, Fisher’s least significant difference (LSD) was calculated at the 5% level to compare means for significant effects, except for light intensity that was calculated at the 10% level [78]. Correlations between variables were established using Pearson correlation coefficients (PCCs) [79]. Treatment effects, seasonal effects, as well as interactions between treatment and season for all discussed variables, except for soil characteristics, are presented in Table S1.

## 5. Conclusions

The micro-climatic conditions in the vineyard did not have pronounced effects on physiological variables. Regardless of the climatic conditions, reduced water supply had noticeable effects on Pn and Tr and resulted in a lower plant water status. The different levels of water supply affected stomatal control. The rates of photosynthesis and transpiration of vines were increased under OPC conditions, compared to OF conditions. Yet, vines grown under OF conditions used water more efficiently. Compared to OF conditions, OPC increased the level of plant tolerance to abiotic stress conditions (such as water deficit). Vines of the two highest water deficit treatments, W070 and W055, were not as stressed in the OPC trial compared to those in the OF trial. Stem water potential reflected the lower plant water status caused by decreased water supply.

The changes that occurred in the plastic product from one season to the next emphasised the importance of the quality of the plastic products in terms of durability, but also with respect to the transmittance and reflectance properties to create optimal micro-climatic conditions, especially light and temperature. The use of an overhead plastic cover to temper and even mitigate the effects of extreme temperatures is a cultivation practice to consider when managing table grape production in less-than-ideal environments. Measurement of stem water potential can be used as an indicator to classify water deficit in different micro-climatic environments. 

The application of W080 was sufficient for photosynthesis and related processes under OF conditions, whereas the application of W070 was sufficient for photosynthesis and related processes under OPC conditions. Photosynthesis was not impaired by the lower light intensity underneath the OPC. Despite higher Pn underneath the OPC compared to the OF conditions, water use efficiency could not be improved by OPC. The measurement of stem water potential proved to be a reliable tool to determine grapevine water status, integrating the effects of the soil, vine, and atmospheric conditions.

## Figures and Tables

**Table 1:** Soil characteristics at 30 cm and 60 cm soil depths of the open field (OF) and overhead plastic covering (OPC) trials in a Crimson Seedless vineyard near Robertson, South Africa (April 2019).

	Trt	Clay (%)		Silt (%)		Sand (%)		Stone (%)		WHC (mm/m)		Base Saturation (%) Ca		Base Saturation (%) Mg		Exchangeable K (cmol/kg)		pH_KCl_	
		Depth (cm)																	
		30	60	30	60	30	60	30	60	30	60	30	60	30	60	30	60	30	60
OF	W100	6.60 ab	6.60 a	2.00 a	1.60 b	91.40 ab	91.80 a	1.60 a	1.34 a	88.55 b	90.32 b	78.38 ab	59.99 a	12.65 ab	16.81 a	0.12 b	0.11 b	6.30 a	5.48 a
	W080	5.40 b	5.80 a	2.80 a	2.00 b	91.80 a	92.20 a	0.60 a	0.86 a	92.43 ab	92.09 b	82.00 a	56.58 a	12.45 b	30.27 a	0.09 b	0.09 b	6.30 a	6.02 a
	W070	7.50 a	7.00 a	3.00 a	3.50 a	89.50 b	89.50 b	1.00 a	1.00 a	105.95 a	111.19 a	68.06 b	64.90 a	19.04 a	17.40 a	0.24 a	0.20 a	6.00 a	5.80 a
	W055	6.00 ab	6.33 a	2.00 a	2.00 b	92.00 a	91.67 a	0.60 a	1.12 a	90.58 b	93.57 ab	79.02 ab	54.30 a	13.81 ab	20.64 a	0.13 b	0.11 b	6.63 a	5.53 a
	LSD (*p* = 0.05)	1.76	1.22	1.18	1.06	2.08	1.93	2.16	2.21	15.10	19.00	12.96	33.38	6.40	21.16	0.09	0.06	0.83	1.43
OPC	W100	6.50 a	7.50 a	2.50 a	2.50 a	91.00 a	90.00 a	0.83 a	0.98 a	103.30 a	98.02 a	70.93 a	47.60 a	21.59 a	24.29 a	0.14 a	0.19 a	6.35 a	5.60 a
	W080	6.50 a	7.50 a	2.0 a	1.50 a	91.50 a	91.00 a	0.40 a	0.38 a	110.46 a	110.61 a	74.45 a	56.04 a	19.78 a	23.02 a	0.14 a	0.16 a	6.53 a	5.45 a
	W070	6.50 a	8.00 a	2.0 a	3.00 a	91.50 a	89.00 a	0.40 a	0.63 a	96.53 a	111.63 a	75.88 a	35.90 a	18.20 a	40.28 a	0.12 a	0.17 a	6.35 a	5.58 a
	W055	7.50 a	8.00 a	1.0 a	2.50 a	91.50 a	89.50 a	0.48 a	1.28 a	104.16 a	103.59 a	73.34 a	57.19 a	18.93 a	24.14 a	0.13 a	0.17 a	6.30 a	5.75 a
	LSD (*p* = 0.05)	1.77	3.15	2.28	2.40	1.87	3.70	0.76	1.32	17.67	14.02	11.32	29.98	6.78	28.31	0.07	0.10	0.44	1.29

Trt = treatment; W = water: W100 (control) (100% = commercial practice)**,** W080 (80% of control), W070 (70% of control), W055 (55% of control). WHC = water holding capacity. Values with the same letter in a column do not differ significantly from each other at the 5% significance level. LSD = Least significant difference.

**Table 2:** Actual irrigation volumes for treatments applied in the open field and overhead plastic covering trials near Robertson, South Africa, over four seasons: 2019/2020 (11 November 2019–26 February 2020); 2020/2021 (23 October 2020–26 February 2021), 2021/2022 (21 October 2021–2 March 2022) and 2022/2023 (11 October 2022–23 February 2023).

		Season							
		2019/2020		2020/2021		2021/2022		2022/2023	
Micro-Climate	Water Treatment	Volume (m^3^/ha)	(%)	Volume (m^3^/ha)	(%)	Volume (m^3^/ha)	(%)	Volume (m^3^/ha)	(%)
Open Field	W100	5089	100	6899	100	5772	100	6387	100
	W080	3859	76	5362	78	4747	82	5260	82
	W070	3381	66	4577	66	4235	73	4406	69
	W055	2698	53	3620	53	3074	53	3313	52
Overhead plastic covering	W100					6045	100	6592	100
	W080					4611	76	5123	78
	W070					4064	67	4508	68
	W055					3313	55	3586	55

W = water treatment: W100 (control) (100% = commercial practice), W080 (80% of control), W070 (70% of control), W055 (55% of control).

**Table 3:** Seasonal effects of water treatments on soil water content of Crimson Seedless vines grown under open field conditions and overhead plastic covering.

Climate		Open Field					Overhead Plastic Covering		
		Season					Season		
Variable	DS *	19/20	20/21	21/22	22/23	LSD (*p* = 0.05)	21/22	22/23	LSD (*p* = 0.05)
Soil water content (%)	2		5.15 c	6.73 b	10.06 a	0.88			
	3	6.22 b	4.78 c	8.97 a	5.97 b	0.58	11.34 a	7.62 b	0.92
	4	4.24 c	4.06 c	10.55 a	6.77 b	1.02	6.08 b	8.33 a	1.23
	5	4.23 c	3.81 c	5.48 b	17.29 a	1.12	7.81 b	20.81 a	2.43
	6	3.08 b		3.93 a	.	0.38			
	7	4.58 b	3.68 b	9.11 a	7.77 a	1.61	3.02 b	9.48 a	1.30
	8	4.55 a	3.77 b			0.35			
	9	4.47 c	5.07 b	4.34 c	6.68 a	0.53	5.73 b	9.38 a	0.64
	10	3.97 c	4.57 b	3.95 c	7.07 a	0.45	5.00 b	9.77 a	0.81
	11	3.93 c	4.84 b	4.35 c	6.70 a	0.46	5.18 b	8.76 a	0.92

* DS = Development stages: 2 = 6 to 11 DAFB (Days after full bloom), 3 = 20 to 25 DAFB), 4 = 34 to 39 DAFB, 5 = 48 to 53 DAFB, 6 = 62 DAFB, 7 = 67 to 77 DAFB, 8 = 78 to 81 DAFB, 9 = 83 to 88 DAFB, 10 = 90 to 95 DAFB, 11 = 97 to 102 DAFB. Values with the same letter per row per development stage do not differ significantly from each other at the 5% significance level. LSD = Least significant difference.

**Table 4:** The effect of water treatments under open field (OF) conditions on air temperature, relative humidity, and solar variables in the micro- (MIC), meso- (MES), and macro-climatic (MAC) environments of a Crimson Seedless vineyard near Robertson, South Africa (2020/2021).

		Development Stage *																				
		4			5			7			8			9			10			11		
	Trt	Climate																				
		MIC	MES	MAC	MIC	MES	MAC	MIC	MES	MAC	MIC	MES	MAC	MIC	MES	MAC	MIC	MES	MAC	MIC	MES	MAC
		OF			OF			OF			OF			OF			OF			OF		
Average temperature (°C)	**W100**	22.72 a	22.08	23.43	23.24 a	23.07	24.32	23.91 a	23.57	25.35	23.32 b	23.24	24.76	24.10 b	24.16	25.68	24.69 b	24.65	26.23	20.83 a	20.76	21.69
	**W080**	22.95 a			23.41 a			24.01 a			23.48 ab			24.27 ab			24.84 ab			20.95 a		
	**W070**	23.16 a			23.59 a			24.05 a			23.51 ab			24.30 ab			24.84 ab			20.92 a		
	**W055**	23.49 a			23.78 a			24.42 a			23.94 a			24.77 a			25.28 a			21.29 a		
LSD (*p* = 0.05)		1.42			0.61			0.55			0.56			0.55			0.55			0.49		
Maximum temperature (°C)	**W100**	23.68 a	30.43	32.51	24.28 a	31.93	33.78	24.87 a	31.22	33.24	24.34 b	31.95	34.42	25.13 b	33.30	36.05	25.70 a	33.43	35.75	21.83 a	29.38	31.54
	**W080**	24.10 a			24.50 a			25.00 a			24.56 ab			25.34 ab			25.89 a			22.02 a		
	**W070**	24.75 a			24.75 a			25.11 a			24.65 ab			25.43 ab			25.96 a			22.05 a		
	**W055**	24.69 a			25.02 a			25.57 a			25.19 a			26.03 a			26.49 a			22.53 a		
LSD (*p* = 0.05)		1.52			0.86			0.83			0.82			0.81			0.82			0.75		
Minimum temperature (°C)	**W100**	21.99 a	15.18	14.35	22.45 a	15.54	14.85	23.21 a	17.96	17.45	22.54 b	15.85	15.10	23.31 b	16.07	15.31	23.93 a	17.86	16.71	20.04 a	13.23	11.84
	**W080**	22.17 a			22.60 a			23.31 a			22.68 ab			23.46 ab			24.05 a			20.15 a		
	**W070**	22.12 a			22.67 a			23.25 a			22.61 ab			23.40 ab			23.96 a			20.02 a		
	**W055**	22.62 a			22.88 a			23.62 a			23.03 a			23.86 a			24.38 a			20.39 a		
LSD (*p* = 0.05)		1.32			0.51			0.45			0.46			0.46			0.46			0.40		
Average relative humidity (%)	**W100**	59.22 a	64.58	55.11	57.34 ab	61.66	52.26	61.39 ab	66.66	55.65	56.76 ab	60.60	52.25	56.88 ab	60.00	52.26	60.59 ab	64.19	53.96	57.64 ab	61.63	52.04
	**W080**	62.04 a			58.94 a			63.211 a			58.14 a			58.27 a			62.15 a			59.28 a		
	**W070**	57.04 a			54.35 b			58.72 b			54.13 b			54.37 b			58.10 b			55.22 b		
	**W055**	60.25 a			58.80 a			61.66 ab			56.71 ab			56.74 ab			60.73 ab			57.95 ab		
LSD (*p* = 0.05)		16.35			4.05			3.95			3.63			3.70			3.84			3.51		
Maximum relative humidity (%)	**W100**	62.76 a	88.37	84.19	61.32 ab	87.51	82.42	64.73 ab	85.94	80.99	60.85 ab	87.01	83.31	60.92 ab	86.37	83.16	64.14 ab	89.71	85.16	61.78 ab	87.77	83.78
	**W080**	66.04 a			63.20 a			66.79 a			62.45 a			62.60 a			65.99 a			63.76 a		
	**W070**	60.72 a			57.86 b			61.67 b			57.72 b			57.99 b			61.34 b			58.97 b		
	**W055**	64.51 a			64.06 a			65.80 ab			61.61 ab			61.71 ab			65.34 ab			62.93 a		
LSD (*p* = 0.05)		18.82			5.30			5.30			3.91			3.95			4.08			3.85		
Minimum relative humidity (%)	**W100**	55.68 a	39.63	26.02	53.35 ab	34.47	22.11	58.05 a	44.56	30.30	52.67 a	33.89	21.19	52.83 a	34.61	21.36	57.04 a	35.97	22.76	53.50 a	33.16	20.30
	**W080**	58.03 a			54.67 a			59.63 a			53.84 a			53.94 a			58.30 a			54.79 a		
	**W070**	53.37 a			50.85 b			55.76 a			50.54 a			50.75 a			54.86 a			51.47 a		
	**W055**	55.99 a			53.53 ab			57.52 a			51.81 a			51.76 a			56.12 a			52.97 a		
LSD (*p* = 0.05)		14.01			3.56			4.01			3.63			3.79			3.98			3.41		
Total radiation (µmol.m^2^/s)	**W100**	**-**	1440.87	1494.69	**-**	1574.86	1634.53	**-**	1513.56	1492.26	**-**	1471.08	1535.08	**-**	1463.82	1541.24	**-**	1413.62	1471.27	**-**	1306.07	1393.16
	**W080**	**-**			**-**			**-**			**-**			**-**			**-**			**-**		
	**W070**	**-**			**-**			**-**			**-**			**-**			**-**			**-**		
	**W055**	**-**			**-**			**-**			**-**			**-**			**-**			**-**		

* Development stages: 4 = 34 to 39 DAFB, 5 = 48 to 53 DAFB , 7 = 67 to 77 DAFB, 8 = 78 to 81 DAFB, 9 = 83 to 88 DAFB, 10 = 90 to 95 DAFB, 11 = 97 to 105. W = water Trt = treatment: W100 (100% = commercial practice), W080 (80% of commercial practice), W070 (70% of commercial practice), W055 (55% of commercial practice). Values with the same letter per column do not differ significantly from each other at the 5% significance level. LSD = Least significant difference.

**Table 5:** The effect of water treatments under open field (OF) and overhead plastic covering (OPC) conditions on air temperature, relative humidity, and solar variables in the micro- (MIC), meso- (MES), and macro-climatic (MAC) environments of a Crimson Seedless vineyard near Robertson, South Africa (2021/2022).

		Development Stage *															
		7				9				10				11			
	Trt	Climate															
		MIC		MES	MAC	MIC		MES	MAC	MIC		MES	MAC	MIC		MES	MAC
		OF	OPC			OF	OPC			OF	OPC			OF	OPC		
Average temperature (°C)	**W100**	25.09 ab	24.87	24.66	27.22	23.36 ab	23.67	23.80	24.11	24.84 a	24.9	24.63	25.95	22.79 a	22.98	22.59	23.56
	**W080**	24.90 b	23.95			23.16 b	22.82			24.87 a	24.31			22.76 a	22.60		
	**W070**	25.39 a	24.11			23.60 a	22.98			25.12 a	24.47			23.04 a	22.76		
	**W055**	25.26 ab	24.34			23.51 a	23.2			24.95 a	24.67			22.88 a	22.80		
LSD (*p* = 0.05)		0.42				0.35				0.34				0.32			
Maximum temperature (°C)	**W100**	25.18 a	26.37	34.25	37.32	23.81 ab	25.10	33.92	32.90	25.87 a	26.36	33.91	35.16	23.92 a	24.37	31.72	33.48
	**W080**	25.08 a	24.77			23.35 b	23.68			26.00 a	25.18			24.07 a	23.50		
	**W070**	25.61 a	24.82			24.08 a	23.74			26.34 a	25.23			24.44 a	23.58		
	**W055**	25.41 a	25.16			23.97 ab	24.06			26.09 a	25.54			24.19 a	23.72		
LSD (*p* = 0.05)		0.60				0.64				0.64				0.68			
Minimum temperature (°C)	**W100**	25.02 ab	23.99	17.75	17.12	23.04 a	22.78	16.16	15.33	24.12 a	24.02	17.19	16.75	21.93 a	22.11	14.65	13.63
	**W080**	24.83 b	23.22			23.07 a	22.04			24.17 a	23.55			21.90 a	21.79		
	**W070**	25.23 a	23.38			23.19 a	22.19			24.29 a	23.71			22.06 a	21.96		
	**W055**	25.19 a	23.54			23.19 a	22.35			24.19 a	23.82			21.99 a	21.93		
LSD (*p* = 0.05)		0.35				0.26				0.30				0.29			
Average relative humidity (%)	**W100**	63.87 c	62.00	65.40	54.99	61.51 b	58.78	61.68	55.65	61.08 ab	60.6	64.69	55.09	59.37 ab	59.28	64.10	56.10
	**W080**	71.37 a	67.69			67.83 a	64.39			64.20 a	65.17			62.28 a	63.22		
	**W070**	62.94 c	67.70			61.61 b	64.79			60.20 b	65.01			58.48 b	63.09		
	**W055**	67.48 b	63.43			65.13 a	60.37			63.10 ab	61.16			61.32 ab	59.66		
LSD (*p* = 0.05)		3.26				3.48				3.29				3.03			
Maximum relative humidity (%)	**W100**	64.80 c	66.59	88.88	85.99	64.25 b	63.45	87.96	85.61	65.37 b	64.84	92.17	87.30	63.22 b	63.51	91.41	89.11
	**W080**	72.73 a	72.93			70.37 a	70.271			69.34 a	70.417			66.62 a	68.59		
	**W070**	63.89 c	72.89			64.33 b	70.74			64.82 b	70.44			62.45 b	68.63		
	**W055**	68.64 b	67.79			68.72 a	65.01			68.13 ab	65.27			65.65 ab	63.96		
LSD (*p* = 0.05)		3.54				3.70				3.69				3.37			
Minimum relative humidity (%)	**W100**	62.93 c	57.41	36.57	23.98	58.76 b	54.12	32.53	25.68	56.78 ab	56.37	33.60	22.87	55.51 ab	55.04	34.40	23.08
	**W080**	70.01 a	62.45			65.29 a	58.5			59.06 a	59.93			57.94 a	57.86		
	**W070**	61.98 c	62.51			58.88 b	58.83			55.59 b	59.58			54.52 b	57.55		
	**W055**	66.33 b	59.06			61.54 b	55.73			58.07 ab	57.04			57.00 ab	55.36		
LSD (*p* = 0.05)		3.00				3.38				2.90				2.69			
Total radiation (µmol.m^2^/s)	**W100**	637.04	-	1493.545	1524.02	560.56	-	1385.70	1411.26	335.67	-	1275.57	1290.632	138.11	-	1387.53	1402.90
	**W080**	690.73	-			209.06	-			436.54	-			637.18	-		
	**W070**	881.86	-			1028.92	-			799.26	-			753.03	-		
	**W055**	-	504.97			879.09	499.28			1109.06	535.509			962.23	962.23		
Photosynthetic active radiation (µmol.m^2^/s)	**W100**	53.66	-	1513.56	-	50.62	-	1463.82	-	88.23	-	1413.62	-	46.43	-	1306.07	-
	**W080**	55.12	-			103.97	-			63.00	-			119.05	-		
	**W070**	120.15	-			170.90	-			91.52	-			96.43	-		
	**W055**	148.77	39.45			129.86	15.74			186.46	45.95			-	14.15		

* Development stages: 7 = 67 to 77 DAFB, 9 = 83 to 88 DAFB, 10 = 90 to 95 DAFB, 11 = 97 to 105. W = water Trt = treatment: W100 (100% = commercial practice), W080 (80% of commercial practice), W070 (70% of commercial practice), W055 (55% of commercial practice). Values with the same letter per column do not differ significantly from each other at the 5% significance level. LSD = Least significant difference.

**Table 6:** The effect of water treatments under open field (OF) and overhead plastic covering (OPC) conditions on air temperature, relative humidity, and solar variables in the micro- (MIC), meso- (MES), and macro-climatic (MAC) environments of a Crimson Seedless vineyard near Robertson, South Africa (2022/2023).

		Development Stage 2022/2023 *																			
		5				7				9				10				11			
	Trt	Climate																			
		MIC		MES	MAC	MIC		MES	MAC	MIC		MES	MAC	MIC		MES	MAC	MIC		MES	MAC
		OF	OPC			OF	OPC			OF	OPC			OF	OPC			OF	OPC		
Average temperature (°C)	**W100**	22.56 ab	23.58	22.15	24.03	22.28 a	22.95	21.73	23.09	23.84 a	25.33	24.87	26.74	23.07 a	22.31	20.53	23.40	24.24 a	23.99	23.73	26.34
	**W080**	22.20 b	23.29			22.54 a	22.73			24.08 a	25.17			21.85 a	22.09			24.33 a	23.72		
	**W070**	22.76 a	24.00			22.28 a	22.67			24.75 a	25.08			21.36 a	22.10			24.37 a	23.91		
	**W055**	22.50 ab	22.38			22.06 a	21.41			24.60 a	23.93			21.22 a	20.95			24.06 a	22.49		
LSD (*p* = 0.05)		0.47	2.70			2.26				1.05				3.66				0.60			
Maximum temperature (°C)	**W100**	23.63 a	24.55	29.66	31.81	23.29 a	24.38	30.34	31.75	24.89 a	26.82	34.79	35.68	24.10 a	23.74	29.90	32.33	25.33 a	25.44	33.16	36.32
	**W080**	23.11 a	24.08			23.63 a	23.55			25.18 a	26.02			22.93 a	22.94			25.41 a	24.53		
	**W070**	23.94 a	25.00			23.56 a	23.64			26.06 a	26.05			22.71 a	23.07			25.57 a	24.90		
	**W055**	23.56 a	23.03			23.22 a	22.20			25.79 a	24.71			22.42 a	21.74			25.33 a	23.28		
LSD (*p* = 0.05)		1.82	3.69			1.03				1.19				3.59				1.48			
Minimum temperature (°C)	**W100**	21.91 a	22.62	16.90	16.24	21.57 a	22.13	15.62	14.43	23.12 a	24.51	18.28	17.80	22.30 a	21.49	14.01	14.47	23.49 a	23.17	17.21	16.35
	**W080**	21.54 b	22.64			21.79 a	22.02			23.32 a	24.44			21.06 a	21.36			23.52 a	23.01		
	**W070**	21.99 a	23.27			21.45 a	21.90			23.91 a	24.31			20.47 a	21.32			23.55 a	23.15		
	**W055**	21.84 a	21.77			21.32 a	20.72			23.84 a	23.27			20.41 a	20.29			23.25 a	21.84		
LSD (*p* = 0.05)		0.24	2.60			0.84				1.06				3.63				0.51			
Average relative humidity (%)	**W100**	68.97 ab	66.17	73.17	34.89	61.68 a	58.50	63.13	24.49	62.00 a	60.25	63.48	23.21	57.11 a	57.44	65.69	27.53	63.76 a	62.03	66.66	26.54
	**W080**	64.89 b	68.01			61.41 a	61.25			62.35 a	62.24			58.49 a	59.75			62.17 a	63.81		
	**W070**	66.30 b	67.94			59.10 a	61.65			61.74 a	62.64			58.01 a	60.07			61.28 a	64.11		
	**W055**	72.37 a	70.62			62.25 a	64.40			64.49 a	65.42			60.52 a	61.86			65.06 a	67.85		
LSD (*p* = 0.05)		5.72	13.56			3.85				3.87				5.65				5.65			
Maximum relative humidity (%)	**W100**	72.35 ab	70.81	91.76	88.10	66.06 ab	63.99	86.91	87.23	65.99 ab	65.47	88.31	84.16	61.25 ab	62.09	87.86	78.95	67.54 a	66.46	90.14	85.73
	**W080**	68.93 b	73.57			66.30 a	67.82			66.73 ab	68.29			62.66 ab	65.40			66.16 a	68.87		
	**W070**	68.99 b	72.53			60.01 b	67.25			62.51 b	67.81			58.78 b	65.04			64.49 a	68.65		
	**W055**	76.32 a	74.17			67.00 a	69.36			68.98 a	69.75			64.91 a	66.12			69.41 a	71.79		
LSD (*p* = 0.05)		6.84	14.45			6.08				4.26				4.64				8.17			
**Minimum relative humidity (%)**	**W100**	65.58 ab	61.52	50.34	26.25	57.30 a	53.02	34.56	28.97	58.02 a	55.04	31.28	26.49	52.98 a	52.79	37.29	25.34	59.98 a	57.59	36.71	26.33
	**W080**	60.85 b	62.46			56.53 a	54.68			57.97 a	56.20			54.32 a	54.11			58.18 a	58.74		
	**W070**	63.61 b	63.34			56.24 a	56.05			59.06 a	57.47			55.44 a	55.09			58.07 a	59.57		
	**W055**	68.43 a	67.07			57.50 a	59.45			60.00 a	61.08			56.13 a	57.60			60.70 a	63.91		
**LSD (*p* = 0.05)**		4.82	13.12			3.73				4.37				6.74				5.70			
**Total radiation (µmol.m^2^/s)**	**W100**		617.84	1465.13	1397.51	690.21	600.46	1596.58	1542.41	673.90	514.28	1619.71	1410.55	533.56	449.09	1432.40	1349.20	528.96	426.06	1556.15	1401.68
	**W080**	789.81	**-**			762.42	693.71			682.74	636.34			657.96	555.45			**-**	543.21		
	**W070**	811.71	**-**			952.88	862.73			824.76	766.07			768.89	675.53			768.06	659.86		
	**W055**		**-**			1364.86	933.21			1278.115	786.47			1313.89	691.13			1453.80	760.54		
**Photosynthetic active radiation (µmol.m^2^/s)**	**W100**	46.30	16.52	1477.74	**-**	50.89	11.36	1493.54	**-**	50.00	9.88	1385.70	**-**	-	7.30	1275.57	**-**	-	6.80	1387.53	**-**
	**W080**	47.62	-			106.25	7.00			89.74	13.56			47.55	13.89			95.11	12.49		
	**W070**	92.42	-			47.40	26.02			104.17	25.00			133.33	17.26			-	18.45		
	**W055**	-	28.46			102.30	33.03			74.36	35.02			85.12	34.14			113.69	31.50		

* Development stages: 5 = 48 to 53 DAFB, 7 = 67 to 77 DAFB, 9 = 83 to 88 DAFB, 10 = 90 to 95 DAFB, 11 = 97 to 102. W = water Trt = treatment: W100 (100% = commercial practice), W080 (80% of commercial practice), W070 (70% of commercial practice), W055 (55% of commercial practice). Values with the same letter per column do not differ significantly from each other at the 5% significance level. LSD = Least significant difference.

**Table 7:** Radiometric properties of the plastic product used in the overhead plastic covering trial before use (2021) and after use (2023).

		Transmittance (%)				Reflectance (%)	
		Diffuse		Direct			
Wavelength Range	Wavelength (nm)	2021	2023	2021	2023	2021	2023
Solar radiation	200–2500	24.3	49.5	55.6	28.0	17.2	17.9
Photosynthetic active radiation	400–700	38.9	52.1	40.8	22.9	17.4	19.0
Visible light	380–760	38.4	52.0	41.4	23.5	17.4	18.9
Solar infrared	760–2500	7.4	46.5	72.6	33.6	17.0	16.8
Ultraviolet	280–380	47.5	52.1	31.2	14.5	18.6	18.2
Ultraviolet-A	320–380	47.4	52.2	31.3	14.6	18.6	18.2
Ultraviolet-B	280–320	51.2	49.0	27.7	12.0	18.5	16.6
Far infrared	7500–12,500	-	-	33.0	26.8	4.5	3.1

**Table 8:** Seasonal effects of water treatments on light intensity and total leaf area of Crimson Seedless vines grown under open field conditions and overhead plastic covering.

Climate		Open Field					Overhead Plastic Covering		
		Season					Season		
Variable	DS *	19/20	20/21	21/22	22/23	LSD (*p* = 0.05)	21/22	22/23	LSD (*p* = 0.05)
** Light intensity (µmol/m^2^/s)	2		188.45 a	101.72 b	59.88 b	51.82 **	74.06 a	35.92 b	29.03
	3	70.01 b	98.72 a	64.81 b	120.44 a	28.32	37.59 a	32.05 a	8.16
	4	198.46 a	52.68 b	67.36 b	75.75 b	40.22	45.69 a	33.68 a	20.50
	5	165.54 a	195.31 a	98.18 b	161.21 a	41.29	52.20 a	50.37 a	15.72
	6	58.19 b		96.1 a		26.00			
	7	38.30 b	76.52 b	163.46 a	134.41 a	41.95	37.27 a	35.30 a	15.61
	8	96.96 b	304.96 a			57.82			
	9	117.74 b	216.61 a	127.76 b	142.07 b	47.86	43.14 a	23.15 b	11.60
	10	79.24 b	118.14 a	46.14 c	107.63 a	23.69	29.88 a	21.19 a	9.67
	11	86.42 c	151.82 a	138.03 ab	109.31 bc	36.33	42.54 a	9.57 b	13.23
*** Total leaf area (m^2^/vine)	2	38.86 a	24.78 c	28.81 bc	31.01 b	4.37			
	3	40.39 a	32.17 b	37.12 a	36.27 ab	4.3	38.20 a	48.75 a	10.61
	4	25.94 bc	33.09 a	22.84 c	30.82 ab	5.07	36.00 a	39.72 a	7.42
	5	34.65 a	32.16 ab	26.56 b	31.75 ab	5.93	37.82 a	36.97 a	10.38
	6								
	7	25.76 a	20.15 b	29.23 a	27.71 a	3.82	35.46 a	33.85 a	11.55
	8	28.82 a	20.49 b			3.86			
	9	21.83 b	19.18 b	30.32 a	33.90 a	4.55	33.78 b	48.44 a	13.85
	10	22.42 b	19.88 b	25.46 a	28.43 a	3.03	33.46 a	35.74 a	12.02
	11	19.30 c	19.46 c	25.71 b	32.04 a	3.95	30.08 a	36.95 a	11.23

* DS = Development stages: 2 = 6 to 11 DAFB (Days after full bloom), 3 = 20 to 25 DAFB), 4 = 34 to 39 DAFB, 5 = 48 to 53 DAFB, 6 = 62 DAFB, 7 = 67 to 77 DAFB, 8 = 78 to 81 DAFB, 9 = 83 to 88 DAFB, 10 = 90 to 95 DAFB, 11 = 97 to 102 DAFB. ** Values with the same letter per row per development stage do not differ significantly from each other at the 10% significance level. *** Values with the same letter per row per development stage do not differ significantly from each other at the 5% significance level. LSD = Least significant difference.

**Table 9:** Seasonal effects of water treatments on stem water potential and photosynthetic variables of Crimson Seedless vines grown under open field conditions and overhead plastic covering.

Climate		Open Field					Overhead Plastic Covering		
		Season					Season		
Variable	DS *	19/20	20/21	21/22	22/23	LSD (*p* = 0.05)	21/22	22/23	LSD (*p* = 0.05)
Stem water potential (kPa)	2		−666.96 a	−671.09 a	−534.25 b	81.73	−495 a	−422.92 b	24.36
	3	−751.65 a	−665.63 b	−497.92 c	−537.5 c	79.87	−489.58 a	−435.23 b	23.28
	4	−1105.22 a	−627.60 b	−580.21 b	−557.29 b	96.08	−435.23 b	−528.41 a	82.76
	5	−893.88 b	−1011.11 a	−809.09 c	−497.92 d	79.94	−743.18 a	−418.75 b	144.26
	6	−902.08 a	−957.29 a	.	.	95.19			
	7	−383.86 d	−1160.94 a	−1062.50 b	−561.46 c	55.75	−852.08 a	−497.92 b	114.87
	8	−854.69 a	−935.93 a	.	.	95.50			
	9	−902.08 c	−1002.62 b	−1103.13 a	−764.58 d	60.24	−776.04 a	−450.00 b	95.72
	10	−939.58 b	−1032.77 a	−1001.04 ab	−601.04 c	64.25	−758.33 a	−439.58 b	123.75
	11	−921.66 b	−976.56 b	−1179.17 a	−539.58 c	84.63	−1035.42 a	−420.83 b	113.41

* DS = Development stages: 2 = 6 to 11 DAFB (Days after full bloom), 3 = 20 to 25 DAFB), 4 = 34 to 39 DAFB, 5 = 48 to 53 DAFB, 6 = 62 DAFB, 7 = 67 to 77 DAFB, 8 = 78 to 81 DAFB, 9 = 83 to 88 DAFB, 10 = 90 to 95 DAFB, 11 = 97 to 102 DAFB. Values with the same letter per column per development stage do not differ significantly from each other at the 5% significance level. LSD = Least significant difference.

**Table 10:** Seasonal effects of water treatments on photosynthetic variables of Crimson Seedless vines grown under open field conditions and overhead plastic covering.

Climate		Open Field					Overhead Plastic Covering		
		Season					Season		
Variable	DS *	19/20	20/21	21/22	22/23	LSD (*p* = 0.05)	21/22	22/23	LSD (*p* = 0.05)
Net photosynthesis (µmol CO_2_/m^2^/s)	2		10.87 b	11.02 b	15.66 a	1.59	14.35 b	16.35 a	1.82
	3	10.02 b	9.46 b	12.45 a	9.21 b	2.05	14.09 a	14.22 a	2.82
	4	8.64 a		9.97 a	9.89 a	2.67	12.16 a	10.57 a	3.52
	5	7.15 bc	5.76 c	7.8 c	13.22 b	1.86	13.94 a	14.26 a	3.01
	6	6.52 a		6.68 a		1.95			
	7		5.26 b	4.40 b	12.34 a	2.11	9.36 a	12.06 a	4.14
	8	6.44 a	5.11 a			1.82			
	9	8.90 b	9.07 b	5.76 c	11.08 a	1.25	11.93 a	12.62 a	3.44
	10	7. 80 b	9.15 b	4.73 c	11.67 a	1.75	8.85 b	12.25 a	2.50
	11	7.01 c	9.12 b	7.20 c	11.19 a	1.31	11.13 a	12.50 a	2.14
Transpiration rate (mmol H_2_O/m^2^/s)	2		6.43 a	4.33 c	5.51 b	0.61	6.65 a	6.21 a	1.22
	3	3.98 b	3.57 b	5.28 a	4.15 b	0.88	6.51 b	7.87 a	1.04
	4	3.65 b		5.96 a	4.17 b	1.34	7.87 a	4.83 b	2.27
	5	3.72 b	2.20 c	3.73 b	6.29 a	0.81	7.92 a	7.03 a	1.12
	6	2.31 b	3.85 a			0.80			
	7		2.64 b	2.76 b	7.08 a	1.14	6.66 a	7.70 a	2.85
	8	4.46 a	1.8 b			0.94			
	9	4.36 b	3.51 c	2.72 d	6.4 a	0.72	7.88 a	8.60 a	2.31
	10	3.41 b	3.24 b	3.07 b	5.72 a	0.97	6.96 a	6.65 a	1.31
	11	2.91 bc	3.64 b	2.80 c	5.00 a	0.77	6.52 a	7.35 a	1.36
Photosynthesis: transpiration ratio (×1000)	2		1.8 c	2.6 b	2.9 a	0.2	2.1 b	2.7 a	0.2
	3	2.5 ab	2.8 a	2.4 b	2.3 b	0.4	2.2 a	1.8 b	0.2
	4	2.4 a		1.7 b	2.6 a	0.4	1.6 b	2.2 a	0.3
	5	1.9 c	2.5 a	2.1 b	2.1 b	0.2	1.8 a	2.1 a	0.3
	6	2.8 a		1.7 b		0.2			
	7		2.1 a	1.5 b	1.8 b	0.3	1.6 a	1.7 a	0.3
	8	1.4 b	3.0 a			0.4			
	9	2.1 b	2.7 a	2.1 b	1.8 c	0.2	1.5 a	1.5 a	0.3
	10	2.4 b	2.9 a	1.6 d	2.1 c	0.1	1.3 b	1.9 a	0.3
	11	2.5 ab	2.6 ab	2.7 a	2.3 b	0.3	1.9 a	1.8 a	0.3

* DS = Development stages: 2 = 6 to 11 DAFB (Days after full bloom), 3 = 20 to 25 DAFB), 4 = 34 to 39 DAFB, 5 = 48 to 53 DAFB, 6 = 62 DAFB, 7 = 67 to 77 DAFB, 8 = 78 to 81 DAFB, 9 = 83 to 88 DAFB, 10 = 90 to 95 DAFB, 11 = 97 to 102 DAFB. Values with the same letter per column per development stage do not differ significantly from each other at the 5% significance level. LSD = Least significant difference.

**Table 11:** Stem water potential (Ψ_S_) values indicating the degrees of water stress in the open field and overhead plastic covering trials.

Degree of Water Deficit	Ψ_S (kPa)_ Wine Grapes [13]	Ψ_S (kPa)_ Table Grapes [38]
No water deficit	>−600	>−600
Mild (weak) water deficit	−600 to −900	−600 to −800
Moderate water deficit	−900 to −1100	−800 to −1000
Moderate (strong) water deficit to severe water stress	−1100 to −1400	−1000 to −1200
Severe water stress	<−1400	<−1200

## Data Availability

Data are contained within the article and Supplementary Materials.

## Supplementary Materials

The following supporting information can be downloaded at: https://www.mdpi.com/article/10.3390/plants14233579/s1, Table S1. Analyses of variance to test treatment, season, and interaction effects of water treatments for average soil water content, light intensity, leaf area and physiological variables in an open field trial and a trial underneath overhead plastic covering in a Crimson Seedless vineyard near Robertson, South Africa from 2019/2020 to 2022/2023.

## Abbreviations

The following abbreviations are used in this manuscript:

W	Water (irrigation) treatment
OF	Open field
OPC	Overhead plastic covering
SWC	Soil water content
Ψ_S_	Stem water potential
Pn	Net Photosynthesis
Tr	Transpiration rate
DS	Development stage
DAFB	Days after full bloom
ET_c_	Crop evapotranspiration
ET_0_	Reference evaporation rate
K_c_	Crop coefficient
PAR	Photosynthetic active radiation
LSD	Least significant difference
WHC	Water-holding capacity
MAC	Macro-climatic environment
MES	Meso-climatic environment
MIC	Micro-climatic environment
T_max_	Maximum temperature
T_min_	Minimum temperature
T_Ave_	Average temperature
RH_max_	Maximum relative humidity
RH_min_	Minimum relative humidity
RH_ave_	Average relative humidity
PCC	Pearson correlation coefficient
UV	Ultraviolet
R/FR	Red/Far-red
