# Supplementary material for: Physiological Response of Crimson Seedless Table Grape Vines to Controlled Irrigation Conditions in Different Micro-Climatic Environments
Source: Plants (Basel). 2025 Nov 23;14(23):3579. doi: 10.3390/plants14233579 (PMC12694231; doi:10.3390/plants14233579)
Supplement: Supplementary file 1 [file plants-14-03579-s001.zip › plants-3973784-supplementary.pdf]

plants

MDPI

Table S1. Analyses of variance to test treatment, season and interaction effects of water treatments for average soil water content, light intensity, leaf area and physiological variables in an open field trial and a trial underneath overhead plastic covering in a Crimson Seedless vineyard near Robertson, South Africa from 2019/2020 to 2022/2023.

Climate		Open field								Overhead plastic covering							
Variable	DS	Source	Rep	T	Error a	Season	T x S	Error b	Corr. total	Source	Rep	Treatment	Error a	Season	T x S	Error b	Corr. total
Soil water content average (%)	**0**	**DF**	5	3	13	0	0	0	21								
		**MS**	0.0992	3.0458	0.0026	.	.	.									
		P	***<0.0001***	***<0.0001***		**.**	**.**										
	**1**	**DF**	5	3	11	0	0	0	19								
		**MS**	0.2611	6.1921	0.0011	.	.	.									
		P	***<.0001***	***<.0001***		**.**	**.**										
	**2**	**DF**	5	3	15	2	6	33	64	**DF**	3	3	7	0	0	0	13
		**MS**	12.4530	19.4737	2.7368	138.1607	1.0326	2.0244		**MS**	41.2217	5.7748	5.4437	.	.	.	
		P	***0.0100***	***0.0034***		***<0.0001***	***0.7963***			***P***	***0.0133***	***0.4244***		**.**	**.**		
	**3**	**DF**	5	3	15	3	9	56	91	**DF**	3	3	9	1	3	9	28
		**MS**	66.2828	17.6595	8.0684	77.9953	0.9791	0.9515		**MS**	52.0252	11.7120	4.9246	87.0211	1.4324	1.2099	
		P	***0.0007***	***0.1318***		***<0.0001***	***0.4289***			***P***	***0.0026***	***0.1375***		***<0.0001***	***0.3693***		
	**4**	**DF**	5	3	15	3	9	54	89	**DF**	3	3	9	1	3	9	28
		**MS**	62.4922	4.9405	6.6634	192.0780	4.8350	2.8919		**MS**	14.6238	3.3630	2.4477	49.4143	13.4771	2.1047	
		P	***0.0003***	***0.5437***		***<0.0001***	***0.1187***			***P***	***0.0159***	***0.3121***		***0.0009***	***0.0130***		
	**5**	**DF**	5	3	15	3	9	51	86	**DF**	3	3	9	1	3	10	29
		**MS**	40.3333	62.7254	7.7433	877.6002	2.5813	3.3531		**MS**	2.1331	9.0839	13.3842	1222.6577	5.8782	8.8652	
		P	***0.0057***	***0.0019***		***<0.0001***	***0.6444***			***P***	***0.9210***	***0.5868***		***<0.0001***	***0.5934***		
	**6**	**DF**	5	3	15	1	3	15	42	**DF**	3	3	8	0	0	0	14
		**MS**	18.0456	4.1394	3.3581	13.7793	0.0330	0.4477		**MS**	12.4871	6.4182	1.7298	.	.	.	
		P	***0.0050***	***0.3325***		***<0.0001***	***0.9731***			***P***	***0.0115***	***0.0613***		**.**	**.**		

 https://doi.org/10.3390/plants14233579

Climate			Open field								Overhead plastic covering						
**Variable**	**DS**	**Source**	**Rep**	**T**	**Error a**	**Season**	**T x S**	**Error b**	**Corr. total**	**Source**	**Rep**	**Treatment**	**Error a**	**Season**	**T x S**	**Error b**	**Corr. total**
	**7**	**DF**	5	3	15	3	9	57	92	**DF**	3	3	9	1	3	6	25
		**MS**	102.6882	7.9211	3.9209	156.6819	4.7685	7.4826		**MS**	2.7978	0.6216	6.4950	247.5733	3.3052	1.8366	
		P	***<0.0001***	***0.1543***		***<0.0001***	***0.7605***			***P***	***0.7360***	***0.9605***		***<0.0001***	***0.2473***		
	**8**	**DF**	5	3	15	1	3	17	44								
		**MS**	18.9325	7.9268	2.1768	12.9422	0.0267	0.3057									
		P	***0.0005***	***0.0374***		***<0.0001***	***0.9661***										
	**9**	**DF**	5	3	14	3	9	49	83	**DF**	3	3	8	1	3	8	26
		**MS**	36.6524	15.1309	4.3403	29.0967	1.1975	0.7274		**MS**	19.6296	5.8463	2.0875	84.4774	0.9154	0.5165	
		P	***0.0007***	***0.0446***		***<0.0001***	***0.1284***			***P***	***0.0053***	***0.1085***		***<0.0001***	***0.2300***		
	**10**	**DF**	5	3	15	3	9	53	88	**DF**	3	3	9	1	3	9	28
		**MS**	37.5084	11.7347	4.6767	48.5535	1.6271	0.5588		**MS**	22.7157	9.1861	5.8709	124.6234	1.1149	0.9207	
		**P**	**0.0007**	**0.0983**		**<0.0001**	**0.0071**			**P**	**0.0498**	**0.2646**		**<0.0001**	**0.3605**		
	**11**	**DF**	5	3	15	3	9	48	83	**DF**	3	3	9	1	3	9	28
		**MS**	34.5645	17.9808	4.5586	23.1350	1.0048	0.5421		**MS**	17.8239	10.6361	4.5145	63.8417	2.1423	1.1955	
		P	***0.0010***	***0.0294***		***<0.0001***	***0.0826***			***P***	***0.0475***	***0.1399***		***<0.0001***	***0.2186***		
**Light intensity (µmol/m/s)**	**0**	**DF**	3	3	9				15								
		**MS**	12788.40	344.10	1436.67												
		P	***0.0047***	***0.8700***													
	**1**	**DF**	3	3	9				15								
		**MS**	12205.18	4310.79	3904.06												
		P	***0.0800***	***0.4000***													
	**2**	**DF**	3	3	9	2	6	20	43	**DF**	3	3	6	1	3	4	20
		**MS**	52651.06	6315.95	7102.10	40747.23	1890.26	6500.20		**MS**	7040.70	196.42	723.23	3608.31	930.04	953.55	
		P	***0.0100***	***0.4800***		***0.0100***	***0.9300***			***P***	***0.0101***	***0.8440***	***0.6377***	***0.1236***	***0.4875***		
	**3**	**DF**	3	3	9	3	9	28	55	**DF**	2	3	6	1	3	8	23
		**MS**	12694.01	6385.17	830.80	12064.57	853.20	1900.12		**MS**	1323.47	70.56	235.23	183.98	192.45	115.46	

Climate		Open field								Overhead plastic covering							
**Variable**	**DS**	**Source**	**Rep**	**T**	**Error a**	**Season**	**T x S**	**Error b**	**Corr. total**	**Source**	**Rep**	**Treatment**	**Error a**	**Season**	**T x S**	**Error b**	**Corr. total**
		P	***0.0007***	***0.0100***		***0.0020***	***0.9000***			***P***	***0.0400***	***0.8200***	***0.1700***	***0.2400***	***0.2500***		
	**4**	**DF**	3	3	9	3	9	28	55	**DF**	2	3	6	1	3	7	22
		**MS**	37707.86	16607.80	7828.00	59378.55	2597.00	3832.10		**MS**	854.96	396.81	909.11	1066.88	298.64	671.66	
		P	***0.0300***	***0.1700***		***<0.0001***	***0.7200***			***P***	***0.4400***	***0.7400***	***0.3500***	***0.2500***	***0.7300***		
	**5**	**DF**	3	3	9	3	9	27	54	**DF**	2	3	6	1	3	7	22
		**MS**	110553.02	24570.57	9148.25	9296.23	5929.16	3972.01		**MS**	804.16	7205.44	2943.43	496.69	1157.84	459.62	
		**P**	***0.0017***	***0.1100***		***0.1000***	***0.2000***			***P***	***0.7700***	***0.1600***	***0.0100***	***0.3300***	***0.1400***		
	**6**	**DF**	3	3	9	1	3	7	26	**DF**	2	3	6	0	0	0	11
		**MS**	351.60	2021.33	555.76	11090.95	3365.80	1227.53		**MS**	3760.35	313.33	864.99	.	.	.	
		P	***0.6100***	***0.0600***		***0.0200***	***0.1200***			***P***	***0.0700***	***0.7800***	**.**	**.**	**.**		
	**7**	**DF**	3	3	9	3	9			**DF**	2	3	6	1	3	8	23
		**MS**	19714.22	24843.65	7341.08	47792.92	2324.86			**MS**	33.11	1018.77	517.56	23.16	109.56	422.89	
		P	***0.1100***	***0.0700***	***0.1200***	***<0.0001***	***0.8100***			***P***	***0.9400***	***0.2200***	***0.3800***	***0.8200***	***0.8500***		
	**8**	**DF**	3	3	9	1	3	12	31								
		**MS**	15986.14	44775.62	5609.95	346140.00	17734.99	8418.07									
		P	***0.1000***	***0.0100***	***0.7200***	***<0.0001***	***0.1500***										
	**9**	**DF**	3	3	9	3	9	28	55	**DF**	2	3	6	1	3	8	23
		**MS**	69495.11	30323.69	11989.92	27144.17	3484.76	5428.64		**MS**	550.71	1511.98	830.31	2397.92	301.93	233.50	
		P	***0.0200***	***0.1200***	***0.0500***	***0.0100***	***0.7500***			***P***	***0.5500***	***0.2400***	***0.0500***	***0.0100***	***0.3400***		
	**10**	**DF**	3	3	9	3	9	27	54	**DF**	2	3	6	1	3	8	23
		**MS**	13882.71	8865.26	1233.54	11720.10	1596.61	1307.68		**MS**	68.07	380.49	135.71	453.05	490.28	162.23	
		P	***0.0021***	***0.0100***	***0.5100***	***0.0003***	***0.3200***			***P***	***0.6300***	***0.1300***	***0.5700***	***0.1300***	***0.0900***		
	**11**	**DF**	3	3	9	3	9	28	55	**DF**	2	3	6	1	3	8	23
		**MS**	29538.19	39495.19	4810.89	17564.54	5194.69	3126.84		**MS**	29.25	852.71	390.90	6523.39	308.15	303.68	
		**P**	***0.0100***	***0.0100***	***0.1800***	***0.00***	***0.1500***			***P***	***0.9300***	***0.1900***	***0.3600***	***0.00***	***0.4400***		
**Total leaf area (m^2)**	**0**	**DF**	5	3	12	0	0	0	20								

**Climate**		**Open field**								**Overhead plastic covering**							
**Variable**	**DS**	**Source**	**Rep**	**T**	**Error a**	**Season**	**T x S**	**Error b**	**Corr. total**	**Source**	**Rep**	**Treatment**	**Error a**	**Season**	**T x S**	**Error b**	**Corr. total**
		**MS**	61.7044312	38.79502	28.5891	.	.	.									
		P	***0.1276***	***0.3027***	**.**	**.**	**.**										
	**1**	**DF**	5	3	15	1	3	15	42								
		**MS**	65.5786045	88.99343	29.1011	906.2164447	20.3129	10.9983									
		P	***0.1023***	***0.0607***	***0.0345***	***<0.0001***	***0.1820***										
	**2**	**DF**	5	3	15	3	9	52	87	**DF**	2	3	6	0	0	0	11
		**MS**	138.583335	590.3303	104.645	869.090779	138.0315	51.7067		**MS**	58.93	112.41	32.35	.	.	.	
		P	***0.3065***	***0.0086***	***0.0312***	***<.0001***	***0.0126***			***P***	***0.2004***	***0.0900***	**.**	**.**	**.**		
	**3**	**DF**	5	3	15	3	9	52	87	**DF**	3	3	7	1	3	7	24
		**MS**	499.592583	303.7866	42.6635	227.472593	72.6682	50.2858		**MS**	69.78	397.60	212.00	503.53	297.70	125.56	
		P	***<0.0001***	***0.0034***	***0.6219***	***0.0068***	***0.1937***			***P***	***0.8000***	***0.2200***	***0.2500***	***0.0900***	***0.1600***		
	**4**	**DF**	5	3	15	3	9	52	87	**DF**	3	3	7	1	3	7	24
		**MS**	193.915672	540.9022	65.5206	456.884702	117.1203	69.991		**MS**	182.76	234.84	64.85	23.20	133.48	61.42	
		P	***0.0470***	***0.0018***	***0.5319***	***0.0008***	***0.1194***			***P***	***0.1200***	***0.0700***	***0.4700***	***0.5600***	***0.1800***		
	**5**	**DF**	5	3	15	3	9	51	86	**DF**	3	3	6	1	3	9	25
		**MS**	401.682283	722.2353	101.858	251.02174	71.27143	94.4654		**MS**	90.15	291.28	160.95	4.67	77.92	136.80	
		P	***0.0175***	***0.0034***	***0.3986***	***0.0581***	***0.6579***			***P***	***0.6600***	***0.2500***	***0.4000***	***0.8600***	***0.6500***		
	**6**	**DF**	5	3	12	0	0	0	20	**DF**	3	3	6	0	0	0	12
		**MS**	256.90623	221.6474	44.5685	.	.	.		**MS**	159.22	528.69	100.85	.	.	.	
		P	***0.0061***	***0.0181***	**.**	**.**	**.**			***P***	***0.2900***	***0.0400***	**.**	**.**	**.**		
	**7**	**DF**	5	3	15	3	9	52	87	**DF**	3	3	6	1	3	9	25
		**MS**	138.8388102	291.4053	100.295	349.838368	58.47868	39.6214		**MS**	54.98	288.28	402.97	16.83	79.46	169.29	
		P	***0.2852***	***0.0692***	***0.0068***	***<0.0001***	***0.1817***			***P***	***0.9300***	***0.5800***	***0.1200***	***0.7600***	***0.7100***		
	**8**	**DF**	5	3	15	1	3	17	44								
		**MS**	60.6525369	276.6985	44.4242	935.5984024	26.00287	37.4562									
		P	***0.2918***	***0.0058***	***0.3646***	***0.0001***	***0.5682***										

**Climate**																	
**Variable**	**DS**	**Source**	**Rep**	**T**	**Error a**	**Season**	**T x S**	**Error b**	**Corr. total**	**Source**	**Rep**	**Treatment**	**Error a**	**Season**	**T x S**	**Error b**	**Corr. total**
			**Open field**								**Overhead plastic covering**						
	**9**	**DF**	5	3	15	3	9	53	88	**DF**	3	3	6	1	3	9	25
		**MS**	148.6739725	233.949	63.978	1104.418842	133.7191	57.2538		**MS**	42.49	485.12	391.95	1398.56	127.59	243.76	
		P	***0.0944***	***0.0369***	***0.3646***	***<0.0001***	***0.0268***			***P***	***0.9500***	***0.3800***	***0.2500***	***0.0400***	***0.6800***		
	**10**	**DF**	5	3	15	3	9	49	84	**DF**	3	3	6	1	3	6	22
		**MS**	37.2897019	57.0565	29.0398	277.1224188	28.78769	24.1223		**MS**	135.39	415.44	481.16	2.21	19.02	138.46	
		P	***0.3217***	***0.1627***	***0.3009***	***<0.0001***	***0.3205***			***P***	***0.8400***	***0.5100***	***0.0800***	***0.9000***	***0.9300***		
	**11**	**DF**	5	3	15	3	9	48	83	**DF**	3	3	7	1	3	5	22
		**MS**	142.6293736	150.7014	74.6377	597.358041	49.77184	40.3839		**MS**	26.70	584.42	47.10	123.14	123.52	109.55	
		P	***0.1521***	***0.1545***	***0.0548***	***<0.0001***	***0.2982***			***P***	***0.6500***	***0.0034***	***0.8500***	***0.3400***	***0.4200***		
**Stem water potential (kPa)**	**0**	**DF**	3	3	9	0	0	0	15								
		**MS**	14351.24	13139.29	2573.14	.	.	.									
		P	***0.0200***	***0.0200***		**.**	**.**	**.**									
	**1**	**DF**	3	3	9	0	0	0	15								
		**MS**	24349.43	37538.14	12492.77	.	.	.									
		P	***0.1900***	***0.0900***		**.**	**.**	**.**									
	**2**	**DF**	3	3	9	2	6	18	41	**DF**	3	3	7	1	3	4	21
		**MS**	39797.49	85012.74	27341.60	57875.58	8030.94	10447.29		**MS**	746.33	2346.35	1753.91	49228.52	1442.06	419.92	
		P	***0.2900***	***0.0800***		***0.0100***	***0.6000***			***P***	***0.7400***	***0.3400***		***0.0004***	***0.1300***		
	**3**	**DF**	3	3	9	3	9	27	54	**DF**	2	3	6	1	3	7	22
		**MS**	99524.73	88641.80	10281.70	117067.45	15882.84	10244.32		**MS**	8521.57	2264.66	462.72	15688.92	6988.24	556.18	
		P	***0.0035***	***0.0100***		***<0.0001***	***0.1800***			***P***	***0.0027***	***0.0500***		***0.0011***	***0.0033***		
	**4**	**DF**	3	3	9	3	9	28	55	**DF**	2	3	6	1	3	6	21
		**MS**	112806.30	109245.82	25852.30	929697.17	20314.05	15086.54		**MS**	8261.42	6770.24	7388.54	57781.25	2323.78	6291.23	
		P	***0.0371***	***0.0402***		***<0.0001***	***0.2587***			***P***	***0.3900***	***0.4900***		***0.0200***	***0.7800***		
	**5**	**DF**	3	3	9	3	9	25	52	**DF**	2	3	6	1	3	7	22
		**MS**	181568.54	35081.70	9977.65	522147.21	9846.11	9796.21		**MS**	51179.21	33533.14	16364.47	669382.10	20288.43	21359.75	

Climate			Open field							Overhead plastic covering							
**Variable**	**DS**	**Source**	**Rep**	**T**	**Error a**	**Season**	**T x S**	**Error b**	**Corr. total**	**Source**	**Rep**	**Treatment**	**Error a**	**Season**	**T x S**	**Error b**	**Corr. total**
		P	***0.0004***	***0.0600***		***<0.0001***	***0.4600***			***P***	***0.1174***	***0.2086***		***0.0008***	***0.4668***		
	**6**	**DF**	3	3	9	1	3	8	27	**DF**	2	3	5	0	0	0	10
		**MS**	3711.59	148048.56	37474.66	27506.51	544.70	11685.38		**MS**	27198.15	114487.85	3604.17	.	.	.	
		P	***0.9600***	***0.0500***		***0.1600***	***0.9900***			***P***	***0.0300***	***0.0011***		**.**	**.**		
	**7**	**DF**	3	3	9	3	9	28	55	**DF**	2	3	6	1	3	8	23
		**MS**	13490.37	93424.30	7844.99	2121481.70	11539.70	5078.78		**MS**	31171.88	61822.92	27552.08	752604.17	5156.25	14889.32	
		P	***0.2300***	***0.0017***		***<0.0001***	***0.0500***			***P***	***0.3800***	***0.1800***		***0.0001***	***0.7900***		
	**8**	**DF**	3	3	9	1	3	12	31								
		**MS**	72131.82	239308.58	9296.09	52804.38	41477.22	15368.39									
		P	***0.0100***	***<0.0001***		***0.0900***	***0.0900***										
	**9**	**DF**	3	3	9	3	9	28	55	**DF**	2	3	6	1	3	8	23
		**MS**	47859.52	470514.25	21399.40	256653.18	16663.95	5929.37		**MS**	8404.95	84884.98	10496.96	637819.01	28600.26	10338.54	
		P	***0.1500***	***0.0002***		***<0.0001***	***0.0200***			***P***	***0.4900***	***0.0200***		***<0.0001***	***0.1100***		
	**10**	**DF**	3	3	9	3	9	27	54	**DF**	2	3	6	1	3	8	23
		**MS**	246878.27	235163.20	29846.56	435571.28	15582.90	6628.33		**MS**	8229.17	77855.90	7725.69	609609.38	10876.74	17278.65	
		P	***0.0100***	***0.0100***		***<0.0001***	***0.0400***			***P***	***0.4000***	***0.0100***		***0.0003***	***0.6200***		
	**11**	**DF**	3	3	9	3	9	27	54	**DF**	2	3	6	1	3	23	38
		**MS**	213198.89	529134.68	29251.73	824256.31	28755.35	11502.48		**MS**	20644.53	188984.38	6269.53	########	62803.82	14511.72	
		**P**	***0.0100***	***0.0004***		***<0.0001***	***0.0300***			***P***	***0.1100***	***0.0005***		***<0.0001***	***0.0400***		
**Photosynthetic rate ($\mu mol\ CO_2/m^2/s$)**	**0**	**DF**	3	3	9	0	0	0	15								
		**MS**	5.4318865	6.005168	2.84136	.	.	.									
		P	***0.1982***	***0.1687***	**.**	**.**	**.**										
	**1**	**DF**	3	3	9	0	0	0	15								
		**MS**	5.69509373	11.43225	2.68195	.	.	.									
		P	***0.1674***	***0.0393***	**.**	**.**	**.**										
	**2**	**DF**	4	3	12	2	6	12	39	**DF**	3	3	7	1	3	3	20

Climate		Open field								Overhead plastic covering							
**Variable**	**DS**	**Source**	**Rep**	**T**	**Error a**	**Season**	**T x S**	**Error b**	**Corr. total**	**Source**	**Rep**	**Treatment**	**Error a**	**Season**	**T x S**	**Error b**	**Corr. total**
		**MS**	4.21887443	4.746155	4.73325	89.2797668	2.054625	3.47427		**MS**	7.23	1.41	8.63	5.17	0.68	1.68	
		P	***0.4985***	***0.4251***	***0.3003***	***<0.0001***	***0.7319***			***P***	***0.5100***	***0.9200***	***0.1000***	***0.1800***	***0.7600***		
	**3**	**DF**	3	3	9	3	9	26	53	**DF**	2	3	6	1	3	8	23
		**MS**	16.14347615	16.29262	5.07145	25.18021643	6.76311	6.63073		**MS**	24.62	15.90	3.93	0.10	5.97	8.98	
		P	***0.0774***	***0.0759***	***0.6489***	***0.0221***	***0.4503***			***P***	***0.0300***	***0.0700***	***0.8300***	***0.9200***	***0.6000***		
	**4**	**DF**	3	3	9	2	6	16	39	**DF**	2	3	6	1	3	8	23
		**MS**	35.1560075	5.949167	5.76727	0.0395725	13.60131	10.3778		**MS**	90.21	11.68	8.53	15.07	4.67	13.98	
		P	***0.0150***	***0.4239***	***0.8131***	***0.9962***	***0.3081***			***P***	***0.0100***	***0.3400***	***0.7200***	***0.3300***	***0.8000***		
	**5**	**DF**	3	3	9	3	9	28	55	**DF**	2	3	6	1	3	8	23
		**MS**	31.65396718	28.31111	6.67331	117.3303378	4.456398	5.62921		**MS**	0.21	11.81	15.04	0.61	10.21	10.21	
		P	***0.0300***	***0.0398***	***0.3420***	***<0.0001***	***0.6264***			***P***	***0.9900***	***0.5400***	***0.3000***	***0.8100***	***0.4400***		
	**6**	**DF**	3	3	9	1	3	8	27	**DF**	2	3	6	0	0	0	11
		**MS**	5.66038216	5.383745	5.52708	0.15089204	20.78892	4.89313		**MS**	4.13	1.77	7.79	.	.	.	
		P	***0.4268***	***0.4468***	***0.4371***	***0.865***	***0.0452***			***P***	***0.61***	***0.87***	**.**	**.**	**.**		
	**7**	**DF**	3	3	9	2	6	15	38	**DF**	2	3	6	1	3	8	23
		**MS**	21.19220718	4.81224	6.93028	191.5116781	4.868978	6.19435		**MS**	0.80	20.19	22.34	43.75	12.60	19.36	
		P	***0.0843***	***0.5783***	***0.4070***	***<0.0001***	***0.5942***			***P***	***0.9700***	***0.4900***	***0.4100***	***0.1700***	***0.6000***		
	**8**	**DF**	3	3	9	1	3	12	31								
		**MS**	2.19870212	1.563605	1.64635	14.23911613	3.503785	5.59701									
		P	***0.3228***	***0.4568***	***0.9628***	***0.1367***	***0.6118***										
	**9**	**DF**	3	3	9	3	9	26	53	**DF**	2	3	6	1	3	8	23
		**MS**	12.62834541	7.70163	2.16005	49.9505822	7.234888	2.40912		**MS**	1.67	11.48	10.79	2.89	1.88	13.36	
		P	***0.0169***	***0.0602***	***0.5419***	***<0.0001***	***0.0136***			***P***	***0.8600***	***0.4300***	***0.5900***	***0.6500***	***0.9300***		
	**10**	**DF**	3	3	9	3	9	28	55	**DF**	2	3	6	1	3	8	23
		**MS**	2.60296354	7.766507	1.46292	101.7819977	6.515253	5.01238		**MS**	3.40	2.23	7.55	69.36	9.78	7.05	
		P	***0.2209***	***0.0222***	***0.9712***	***<0.0001***	***0.2808***			***P***	***0.6600***	***0.8300***	***0.4500***	***0.0100***	***0.3200***		

Climate		Open field								Overhead plastic covering							
Variable	DS	Source	Rep	T	Error a	Season	T x S	Error b	Corr. total	Source	Rep	Treatment	Error a	Season	T x S	Error b	Corr. total
	11	DF	3	3	9	3	9	28	55	DF	2	3	6	1	3	7	22
		MS	17.89929854	5.382241	0.7909	46.4404183	5.63397	2.79692		MS	9.94	14.09	3.28	15.52	4.29	4.69	
		P	***0.0002***	***0.0109***	***0.9741***	***<0.0001***	***0.0757***			*P*	***0.1200***	***0.0600***	***0.6600***	***0.1100***	***0.4800***		
	0	DF	3	3	9	0	0	0	15								
		MS	1.90	2.07	0.69	.	.	.									
		P	0.10	0.09	.	.	.										
	1	DF	3	3	9	0	0	0	15								
		MS	4.42	4.65	0.33	.	.	.									
		P	***0.0012***	***0.0010***	.	.	.										
	2	DF	4	3	12	2	6	12	39	DF	3	3	7	1	3	4	21
		MS	3.63	1.15	1.40	16.22	0.56	0.51		MS	1.03	0.17	0.76	0.25	0.15	1.05	
		P	***0.0900***	***0.5100***	***0.0500***	***<0.0001***	***0.4100***			*P*	***0.3300***	***0.8800***	***0.6700***	***0.6500***	***0.9300***		
	3	DF	3	3	9	3	9	26	53	DF	2	3	6	1	3	8	23
Transpiration rate		MS	3.93	5.34	0.47	5.59	1.37	1.23		MS	13.09	3.94	1.34	11.10	0.78	1.23	
(mmol $H_2O/m^2/s$		*P*	***0.0100***	***0.0020***	***0.9300***	***0.0100***	***0.3900***			*P*	***0.0100***	***0.1200***	***0.4400***	***0.0200***	***0.6100***		
	4	DF	3	3	9	2	6	16	39	DF	2	3	6	1	3	8	23
		MS	10.76	0.69	2.63	12.73	3.62	2.62		MS	4.94	0.57	4.98	55.39	0.37	5.84	
		P	***0.0400***	***0.8500***	***0.4800***	***0.0200***	***0.2800***			*P*	***0.4200***	***0.9500***	***0.5700***	***0.0200***	***0.9800***		
	5	DF	3	3	9	3	9	27	54	DF	2	3	6	1	3	8	23
		MS	7.71	7.74	1.37	32.12	0.96	1.05		MS	16.51	8.19	0.89	4.74	4.21	1.41	
		P	***0.0200***	***0.0200***	***0.2800***	***<0.0001***	***0.5300***			*P*	***0.0027***	***0.0100***	***0.7000***	***0.1000***	***0.1000***		
	6	DF	3	3	9	1	3	8	27	DF	2	3	6	0	0	0	11
		MS	1.02	2.78	1.19	13.58	3.52	0.82		MS	11.29	0.60	0.73	.	.	.	
		P	***0.5000***	***0.1400***	***0.3000***	***0.0036***	***0.0400***			*P*	***0.0043***	***0.5300***	.	.	.		
	7	DF	3	3	9	2	6	15	38	DF	2	3	6	1	3	8	23
		MS	2.14	0.94	2.02	71.15	3.40	1.81		MS	18.67	13.41	18.39	6.53	9.87	9.17	

**Climate**		**Open field**								**Overhead plastic covering**							
**Variable**	**DS**	**Source**	**Rep**	**T**	**Error a**	**Season**	**T x S**	**Error b**	**Corr. total**	**Source**	**Rep**	**Treatment**	**Error a**	**Season**	**T x S**	**Error b**	**Corr. total**
		P	***0.4100***	***0.7100***	***0.4100***	***<0.0001***	***0.1500***			***P***	***0.4200***	***0.5700***	***0.1800***	***0.4200***	***0.4100***		
	**8**	**DF**	3	3	9	1	3	12	31								
		**MS**	1.61	1.13	0.97	56.70	0.42	1.49									
		P	***0.2400***	***0.3800***	***0.7400***	***<0.0001***	***0.8400***										
	**9**	**DF**	3	3	9	3	9	26	53	**DF**	2	3	6	1	3	7	22
		**MS**	2.41	4.97	0.68	26.36	2.14	0.81		**MS**	3.10	7.97	0.57	4.99	4.25	5.49	
		P	***0.0600***	***0.0100***	***0.5800***	***<0.0001***	***0.0200***			***P***	***0.0500***	***0.0041***	***0.9900***	***0.3700***	***0.5400***		
	**10**	**DF**	3	3	9	3	9	28	55	**DF**	2	3	6	1	3	8	23
		**MS**	4.86	3.72	0.32	17.20	1.00	1.53		**MS**	7.62	1.58	1.98	0.58	3.83	1.94	
		P	***0.0007***	***0.0019***	***0.9900***	***<0.0001***	***0.7400***			***P***	***0.0800***	***0.5400***	***0.4700***	***0.6000***	***0.2000***		
	**11**	**DF**	3	3	9	3	9	28	55	**DF**	2	3	6	1	3	8	23
		**MS**	4.20	3.34	0.39	12.26	1.40	0.96		**MS**	25.57	13.55	1.65	4.16	4.96	2.08	
		P	***0.0025***	***0.0100***	***0.9200***	***<0.0001***	***0.2100***			***P***	***0.0043***	***0.0200***	***0.6000***	***0.2000***	***0.1500***		
	**0**	**DF**	3	3	9	0	0	0	15								
		**MS**	1.11401E-07	7.74E-08	8.6E-08	.	.										
		P	***0.3352***	***0.4789***	**.**	**.**	**.**										
	**1**	**DF**	3	3	8	0	0	0	14								
		**MS**	1.76679E-07	1.25E-07	1E-08	.	.	.									
		P	***0.0007***	***0.0022***	**.**	**.**	**.**										
**Photosynthesis:transpiration ratio**	**2**	**DF**	4	3	12	2	6	12	39	**DF**	3	3	7	1	3	4	**21**
		**MS**	1.15597E-06	2.1E-07	6E-08	4.07755E-06	8.11E-09	7E-08		**MS**	6.62E-07	3.03E-08	1.45E-07	6.28E-07	1.83E-08	3.46E-08	
		P	***<0.0001***	***0.0493***	***0.5839***	***<0.0001***	***0.9919***			***P***	***0.0400***	***0.8900***	***0.0900***	***0.0100***	***0.6900***		
	**3**	**DF**	3	3	9	3	9	26	53	**DF**	2	3	6	1	3	8	**23**
		**MS**	3.26458E-07	1.3E-07	1.4E-07	5.89984E-07	1.25E-07	2E-07		**MS**	1.02E-06	1.41E-07	8.79E-08	9.74E-07	1.34E-08	4.38E-08	
		P	***0.1506***	***0.4794***	***0.6796***	***0.0502***	***0.7599***			***P***	***0.0100***	***0.2800***	***0.1800***	***0.0015***	***0.8200***		
	**4**	**DF**	3	3	9	2	6	16	39	**DF**	2	3	6	1	3	8	**23**

Climate	Open field									Overhead plastic covering							
**Variable**	**DS**	**Source**	**Rep**	**T**	**Error a**	**Season**	**T x S**	**Error b**	**Corr. total**	**Source**	**Rep**	**Treatment**	**Error a**	**Season**	**T x S**	**Error b**	**Corr. total**
		**MS**	3.15558E-07	7.46E-08	1.5E-07	2.70361E-06	1.09E-07	1.9E-07		**MS**	1.72E-06	2.74E-07	1.08E-07	2.41E-06	2.00E-07	9.76E-08	
		P	***0.1772***	***0.7012***	***0.5985***	***0.0002***	***0.7336***			***P***	***0.0040***	***0.1500***	***0.4300***	***0.0011***	***0.19***		
	**5**	**DF**	3	3	9	3	9	27	54	**DF**	2	3	6	1	3	8	**23**
		**MS**	1.3283E-07	1.33E-07	1.4E-07	1.08818E-06	1.29E-07	7E-08		**MS**	1.24E-06	1.41E-07	4.98E-08	5.89E-07	5.68E-08	1.36E-07	
		P	***0.4584***	***0.4573***	***0.0773***	***<0.0001***	***0.1047***			***P***	***0.0012***	***0.1300***	***0.8800***	***0.0700***	***0.7500***		
	**6**	**DF**	3	3	9	1	3	8	27	**DF**	2	3	6	0	0	0	**11**
		**MS**	9.09224E-07	7.7E-08	4E-08	6.88225E-06	2.46E-07	6E-08		**MS**	7.94E-07	5.51E-09	7.93E-08	.	.	.	
		P	***0.0001***	***0.1930***	***0.7125***	***<0.0001***	***0.0461***			***P***	***0.0100***	***0.9700***	**.**	**.**	**.**		
	**7**	**DF**	3	3	9	2	6	16	39	**DF**	2	3	6	1	3	8	**23**
		**MS**	7.48762E-07	8.75E-08	4E-07	2.39295E-06	1.72E-07	1.3E-07		**MS**	6.07E-07	1.15E-07	2.53E-07	1.08E-08	1.28E-07	9.92E-08	
		P	***0.2065***	***0.8818***	***0.0217***	***<0.0001***	***0.2938***			***P***	***0.1700***	***0.7200***	***0.1100***	***0.7500***	***0.3400***		
	**8**	**DF**	3	3	9	1	3	12	31								
		**MS**	5.32593E-07	2.66E-07	2.7E-07	0.00001842	1E-08	2.6E-07									
		P	***0.1861***	***0.4395***	***0.4732***	***<0.0001***	***0.9833***										
	**9**	**DF**	3	3	9	3	9	26	53	**DF**	2	3	6	1	3	8	**23**
		**MS**	3.86388E-07	7.57E-07	1.1E-07	1.70428E-06	1.06E-07	5E-08		**MS**	2.33E-07	2.95E-08	1.09E-07	1.11E-08	4.45E-08	9.33E-08	
		P	***0.0604***	***0.0101***	***0.0663***	***<0.0001***	***0.0723***			***P***	***0.2000***	***0.8400***	***0.4100***	***0.7400***	***0.7100***		
	**10**	**DF**	3	3	9	3	9	28	55	**DF**	2	3	6	1	3	8	**23**
		**MS**	1.65198E-06	2.25E-07	7E-08	0.00000338	5E-08	3E-08		**MS**	7.67E-07	1.24E-07	8.96E-08	2.18E-06	8.47E-08	7.61E-08	
		P	***0.0002***	***0.0824***	***0.0354***	***<0.0001***	***0.1219***			***P***	***0.0200***	***0.3300***	***0.4000***	***0.0007***	***0.4000***		
	**11**	**DF**	3	3	9	3	9	28	55	**DF**	2	3	6	1	3	8	**23**
		**MS**	3.59762E-08	9.6E-07	2.5E-07	2.44346E-07	2E-07	1.3E-07		**MS**	6.77E-07	4.33E-07	1.39E-07	6.16E-08	4.85E-07	1.16E-07	
		P	***0.9326***	***0.0528***	***0.0876***	***0.1593***	***0.1874***			***P***	***0.0600***	***0.1100***	***0.3900***	***0.4900***	***0.0500***		

Non-Normality (P<W), DF = Degrees of freedom, P = Probability of F-ratio test, MS = Mean square

*Development stages (DS): 1 = berry set, 2 = 6 to 11 DAFB (Days after full bloom), 3 = 20 to 25 DAFB), 4 = 34 to 39 DAFB, 5 = 48 to 53 DAFB, 6 = 62 DAFB, 7 = 67 to 77 DAFB, 8 = 78 to 81 DAFB, 9 = 83 to 88 DAFB, 10 = 90 to 95 DAFB, 11 = 97-105.

Rep = replicate, T = Treatment, S = Season, Corr = Corrected total
